# Novel metal peroxide nanoboxes restrain *Clostridioides difficile* infection beyond the bactericidal and sporicidal activity

**DOI:** 10.1002/btm2.10593

**Published:** 2023-09-05

**Authors:** Li‐Xing Yang, Yi‐Hsin Lai, Chun In Cheung, Zhi Ye, Tzu‐Chi Huang, Yu‐Chin Wang, Yu‐Cheng Chin, Zi‐Chun Chia, Ya‐Jyun Chen, Meng‐Jia Li, Hsiu‐Ying Tseng, Yi‐Tseng Tsai, Zhi‐Bin Zhang, Kuan‐Hsu Chen, Bo‐Yang Tsai, Dar‐Bin Shieh, Nan‐Yao Lee, Pei‐Jane Tsai, Chih‐Chia Huang

**Affiliations:** ^1^ Department of Photonics National Cheng Kung University Tainan Taiwan; ^2^ School of Dentistry and Institute of Oral Medicine National Cheng Kung University Tainan Taiwan; ^3^ Institute of Basic Medicine National Cheng Kung University Tainan Taiwan; ^4^ Department of Medical Laboratory Science and Biotechnology National Cheng Kung University Tainan Taiwan; ^5^ Center of Applied Nanomedicine and Core Facility Center National Cheng Kung University Tainan Taiwan; ^6^ iMANI Center of the National Core Facility for Biopharmaceuticals National Science and Technology Council Taipei Taiwan; ^7^ Department of Stomatology National Cheng Kung University Hospital Tainan Taiwan; ^8^ Department of Medicine National Cheng Kung University Tainan Taiwan; ^9^ Division of Infectious Diseases, Department of Internal Medicine and Center for Infection Control National Cheng Kung University Hospital Tainan Taiwan; ^10^ Research Center of Infectious Disease and Signaling National Cheng Kung University Tainan Taiwan; ^11^ Department of Pathology, National Cheng Kung University Hospital, College of Medicine National Cheng Kung University Tainan Taiwan

**Keywords:** AgAu, AgCl, *Clostridioides difficile* infection, peroxide, spore

## Abstract

*Clostridioides difficile* spores are considered as the major source responsible for the development of *C*. *difficile* infection (CDI), which is associated with an increased risk of death in patients and has become an important issue in infection control of nosocomial infections. Current treatment against CDI still relies on antibiotics, which also damage normal flora and increase the risk of CDI recurrence. Therefore, alternative therapies that are more effective against *C*. *difficile* bacteria and spores are urgently needed. Here, we designed an oxidation process using H_2_O_2_ containing PBS solution to generate Cl^−^ and peroxide molecules that further process Ag and Au ions to form nanoboxes with Ag–Au peroxide coat covering Au shell and AgCl core (AgAu‐based nanoboxes). The AgAu‐based nanoboxes efficiently disrupted the membrane structure of bacteria/spores of *C*. *difficile* after 30–45 min exposure to the highly reactive Ag/Au peroxide surface of the nano structures. The Au‐enclosed AgCl provided sustained suppression of the growth of 2 × 10^7^ pathogenic *Escherichia coli* for up to 19 days. In a fecal bench ex vivo test and in vivo CDI murine model, biocompatibility and therapeutic efficacy of the AuAg nanoboxes to attenuate CDI was demonstrated by restoring the gut microbiota and colon mucosal structure. The treatment successfully rescued the CDI mice from death and prevented their recurrence mediated by vancomycin treatment. The significant outcomes indicated that the new peroxide‐derived AgAu‐based nanoboxes possess great potential for future translation into clinical application as a new alternative therapeutic strategy against CDI.

**Translational Impact Statement**

Compared to vancomycin treatment, the peroxide‐AgAu nanoboxes exhibit higher biocompatibility, attenuate *Clostridioides difficile* infection (CDI), restore the gut microbiota and colon mucosal structure, and prevent CDI recurrence. This new nanodrug possesses great potential for future translation into clinical application as a new alternative therapeutic strategy against CDI.

## 1 INTRODUCTION

*Clostridioides difficile* is a major causative pathogen of nosocomial infections worldwide, with symptoms ranging from mild diarrhea to life‐threatening pseudomembranous colitis and toxic megacolon. Immunocompromised conditions and the application of broad‐spectrum antibiotics, which can disrupt the normal intestinal microbiota, are some of the major risk factors for the development of *C*. *difficile* infection (CDI).^1^ The incidence of CDI has progressively increased in the United States over the past decade,^2^, ^3^ which places substantial clinical and economic burdens on hospitals.^4^, ^5^ CDI was associated with an increased risk of death, new long‐term care facility transfer, and new short‐term skilled nursing facility transfer.^6^ *C*. *difficile* is frequently transmitted in health care settings via health care workers, so CDI is an important issue in infection control.^7^

*Clostridioides difficile* spores are considered a significant transmissible infection through the fecal‐oral route and are most frequently attributed to the healthcare settings. Patients acquire CDI by oral ingestion of spores that are highly resistant to harsh environmental conditions such as acidic pH, alcohol, extreme temperature, and common chemical detergents.^8^, ^9^, ^10^, ^11^ Antibiotics that alter the gut microbiota are the driving force behind the depletion of secondary bile acid production in the gut. Primary bile acids, including cholate, taurocholate and glycocholate, allow *C*. *difficile* spore germination and colonization in the gut.^12^, ^13^, ^14^ Despite the use of vancomycin and fidaxomicin as standard drugs for the treatment of CDI, clinical recurrence rates remain high. *C*. *difficile* recurrence is the most frequent complication of infection affected by the amount or viability of *C*. *difficile* spores in the gut lumen or reinfection with spores from the environment. The confirmation of CDI recurrence is recognized as gut microbiota disruption coincident with viable *C*. *difficile* spores. Therefore, developing novel and better therapeutic interventions targeted to *C*. *difficile* spores or vegetative cells (bacteria) is imperative and needs to be further investigated.

Previous studies have demonstrated good inhibition of *C*. *difficile* spores by chemical disinfectants such as glutaraldehyde (20 mg/mL), sodium hypochlorite (0.25 mg/mL), H_2_O_2_ (15 mg/mL), and formaldehyde (5 mg/mL).^15^ However, these chemical products are too toxic, causing dermal or tissue irritation, and are acceptable for environmental usage but are not suitable for treatment in patients. Moreover, currently available antibiotics target bacteria rather than spores. In addition, resistance to multiple antibiotics is becoming a common feature of *C*. *difficile* strains.^16^ Therefore, alternative nonantibiotic agents that target both *C*. *difficile* bacteria and spores are needed, and dual‐function materials will shed light on preventing recurrence and avoiding the development of drug resistance in CDI.

Recently, resistance to multiple antibiotics has become a common feature of newly emergent strains, and an increasing number of nanoparticle types are regarded as potential new‐generation broad‐spectrum antimicrobial agents.^17^ For example, carbon‐based,^18^, ^19^, ^20^ metallic,^21^, ^22^, ^23^, ^24^ metal oxides,^25^, ^26^, ^27^ organic modified,^28^, ^29^ and metal–organic framework^30^, ^31^, ^32^ nanomaterials have been demonstrated to exhibit antimicrobial efficacy even in multidrug‐resistant bacteria.^33^, ^34^, ^35^ However, these current antibacterial nanoparticles mainly target to vegetative cells, and the sporicidal activity of these nanoparticles has rarely been explored. Yamaguchi and his coworkers reported that WO_3_ at high concentrations (up to 25 mg/50 mL)^36^ with long‐term (24 h) visible light irradiation could only result in a 2.5‐log reduction in *Bacillus subtilis* (gram‐positive bacteria) spores, probably due to the high rigidity of the spore coats.

**FIGURE 1:**
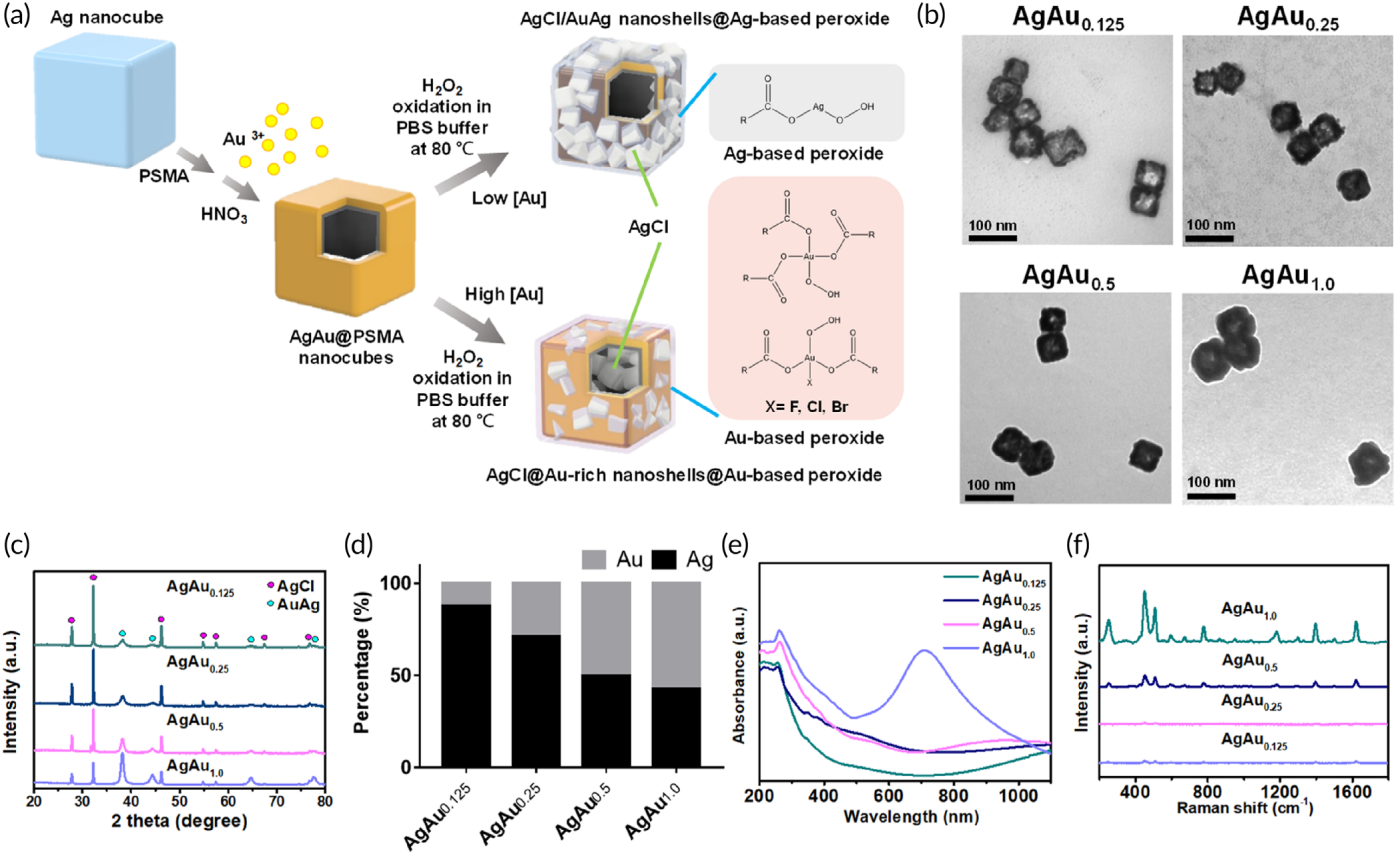
(a) Scheme of the synthesis reactions to fabricate the AgCl/AuAg nanoshells@Ag‐based peroxide (AgAu_0.125_ nanoboxes) and AgCl@Au‐rich nanoshells@Au‐based peroxide (AgAu_1.0_ nanoboxes) using low and high Au ion concentrations in H_2_O_2_‐containing PBS solution. (b) TEM images, (c) XRD pattern, (d) AAS measurements, and (e) UV–visible spectra of the AuAg‐based nanoboxes. (f) Raman spectra of the mixed solution of 0.05 mM methylene blue and 0.5 mM_[Ag]_ AuAg‐based nanoboxes.

Herein, we designed an oxidation process with H_2_O_2_ in PBS solution and the regeneration of Cl^−^ and peroxide molecules as well as Ag–Ag on Ag nanocube template to fabricate Ag–Au nanoboxes. The nanoboxes consisted of peroxide surface and Au shell that enclosed the center AgCl nanoparticle, referred to as AgAu‐based nanoboxes, as shown in Figure 1a. The AgAu‐based nanoboxes efficiently disrupted the membrane structure upon short‐term contact (10–30 min) of the highly active Ag peroxide surface structure with the vegetative cells and the spores of the bacteria. A slow and continuous release of Ag ions from the AgCl interior of AgAu‐based nanoboxes led to sustained suppression of up to 1 × 10^6^–2 × 10^7^ CFUs *Escherichia coli* (gram‐negative bacteria) at 0.5–10 ppm_Ag_ and 10^4^ CFUs *C*. *difficile* (gram‐positive bacteria) at 25 ppm_Ag_. This AgCl‐included structure in AgAu‐based nanoboxes maintained excellent antibacterial efficacy for over 15 days. A strong inhibitory effect on *C*. *difficile* spore germination of up to 72.7% was observed using 100 ppm_Ag_. These antibacterial efficacies were superior to other reported metal oxide nanoparticles,^36^, ^37^, ^38^ Ag‐based nanoparticles, and photodynamic functional nanomaterials. Compared to the rigid structure of bacteria, the high flexibility of the mammalian cell membrane could provide stronger barrier, and the inert Au‐shielding shell can diminish the immediate toxicity to normal cells and probiotics in long‐term contact. The survival of *C*. *difficile* in a fecal bench ex vivo test showed that the nanoboxes exhibited bactericidal ability against *C*. *difficile* in a dose‐dependent manner. In an experimental CDI murine model, we showed that the AuAg‐based nanoboxes exhibited prominent therapeutic efficacy to attenuate CDI.

## 2 MATERIALS AND METHODS

### 2.1 Chemicals

Tannic acid (TNA), methylene blue (MB), hydrogen tetrachloroaurate (III) trihydrate (HAuCl_4_•3H_2_O, 99%) from Alfa Aesar. Ethylene glycol, silver nitrate (HNO_3_) from Fisher Chemical. Polyvinylpyrrolidone (PVP, M.W. ~ 55,000), hydrogen peroxide (H_2_O_2_), iron (III) oxide (γ‐Fe_2_O_3_), silver nitrate (AgNO_3_, 99.8%), poly (styrene‐alt‐maleic acid) sodium salt (PSMA, 13 wt% in H_2_O), and sodium hydroxide (NaOH, 97%) from Sigma‐Aldrich. Iron (II) chloride tetrahydrate (FeCl_2_•4H_2_O, 99%–102%) and sodium citrate dehydrate (99%) from J. T. Baker. Iron (III) chloride hexahydrate (FeCl_3_•6H_2_O) and ammonia solution (25%) from Merck. Hydrochloric acid (HCl, 37%) from Fluka. Sodium iron chlorophyllin salt (Chl/Fe) from JenKem Technology. Sodium borohydride (NaBH_4_, 95%) from Riedel‐de Haen.

### 2.2 Characterizations

Transmission electron microscopy (TEM, Hitachi H7500 TEM instrument at 80 kV) was utilized to determine the structures of the AgAu‐based nanoboxes. The absorption spectra of the AgAu‐based nanoboxes were measured by a V‐730 UV–Vis spectrophotometer from Jasco (USA). The Ag concentrations of AgAu‐based nanoboxes were quantified by AAS (SensAA GBC, Australia). The FT‐IR spectra were obtained by Fourier‐transform infrared spectroscopy (JASCO FT/IR‐4700) with a KBr plate. X‐ray photoelectron spectra (XPS) (PHI 5000 VersaProbe, Japan) were utilized to measure AgAu‐based nanoboxes by a Mg Kα source (12 kV and 10 mA). The binding energy scale was calibrated to the central C 1*s* peak at 284.5 eV. The thin film x‐ray diffractometer (XRD; Bruker D8 Discover, Karlsruhe, Germany) was used to record the crystal structure change of AgAu‐based nanoboxes.

### 2.3 Preparation of Ag nanoplate

A mixture of 0.6 mL of AgNO_3_ (5 mM), 1.8 mL of PVP (0.1 mM), 1.8 mL of sodium citrate (33 mM), and 25 mL of deionized water was prepared. Subsequently, the mixture added 60 μL of H_2_O_2_, 300 μL of NaBH_4_ (100 mM), and 0.1 mL of PSMA (0.02 M). After purification, the final Ag nanoplate was collected and resuspended into 1 mL of deionized water.

### 2.4 Preparation of Ag nanocube

Ag nanocubes were prepared from the single crystalline seed using a NaHS‐mediated polyol reduction process at a high temperature from our previous report.^39^ Briefly, 10 mL of ethylene glycol (EG) solution was first heated to 160°C for 1 h, followed by 0.24 mL of NaHS (3 mM) addition for 2 min under magnetic stirring. Next, 0.5 mL of HCl (3 mM, dispersed in EG) and 2.5 mL of a PVP (20 mg/mL, dispersed in EG) solution were added to the previous solution and reacted for an additional 2 min. Finally, 0.8 mL of CF_3_COOAg (282 mM in EG) was injected into the refluxed mixture and reacted for 1 h. The final solution with greenish‐gray color was quenched in an ice bath, washed with acetone and water three times, and finally dispersed into DI water for further use.

### 2.5 Preparation of AgAu@PSMA nanocubes and AgAu‐based nanoboxes

PSMA (2.5 mL of an 8.9 μM solution) and 250 μL of 12 mM Ag nanocube^39^ were added to a two‐neck round‐bottomed flask with rapid magnetic stirring. Four milliliters of HAuCl_4_ solution (0.125, 0.25, 0.5, or 1 mM) was added into the flask by using a dropwise addition process to get AgAu@PSMA nanocubes. Then, 200 μL of HNO_3_ solution (0.22 M) was added immediately, followed by stirring for another 5 min until the color became stable. Then, 2.5 mL of 1× phosphate‐buffered saline (PBS) and 2.5 mL of 1.4 M H_2_O_2_ were added into the flask and heated in an 80°C oil bath under reflux for 4 min, then quenched in an iced‐water bath. The solution was centrifuged at 7000 rpm for 10 min and followed three times wash to collect AgAu‐based nanoboxes stored in deionized water.

### 2.6 Preparation of Au@MB

Twenty‐five microliters of MB (5 mM) was added into 4.5 mL of HAuCl_4_ (1.1 mM) solution mixed with 0.5 mL of TNA (2.5 mM) to prepare Au@MB.^40^

### 2.7 Preparation of TNA‐coated Fe_3_O_4_

Five milliliters of γ‐Fe_2_O_3_ (0.1 M) dissolved in deionized water was added into 2.25 mL of NaOH solution (50 nM) contained with TNA (0.02 M) and heated at 200°C for 13 h. After cooling and centrifugation, TNA‐coated Fe_3_O_4_ was resuspended in deionized water.^41^

### 2.8 Preparation of Fe_3_O_4_ @Chl

FeCl_3_•6H_2_O (1 M) and Chl/Fe (0.14 M) were dissolved in 2 mL of HCl. Afterward, 0.5 mL of FeCl_2_•4H_2_O (2 M) and 20 mL of NH_4_OH (2 M) were added to the Fe mixture. The Fe_3_O_4_@Chl was dispersed in deionized water after purification.^42^

### 2.9 SERS measurement

The 10 μL of aliquot samples with 0.5 mM_[Ag]_ was dropped on Si substrate for SERS measurement. SERS spectra were acquired from a Jobin–Yvon LabRAM high‐resolution micro‐Raman spectrometer (Horiba iHR 320) with 785 nm laser excitation (DPSSL Driver II), 7 mW of power, 1200 grating number, 10 s of acquisition time and integrated into an Olympus BX53 microscope with ×20 objective lens. Each raw spectrum (200–1800 cm^−1^) was baseline corrected to remove the fluorescence background.

### 2.10 LDI‐MS detection of AgAu‐based nanoboxes

An AutoflexIII LDI time‐of‐flight (TOF/TOF) mass spectrometer (Bruker Daltonics, Bremen, Germany) in positive ion reflectron mode was used, with a SmartBeam laser (Nd:YAG, 355 nm, pulse width 6 ns, pulse duration 200 ns at 100 Hz) serving as the laser source, for the mass spectrometry experiments. The ions produced during laser ablation were stabilized by a delayed extraction period of 10 ns and then accelerated by a voltage ranging from +20 kV to −20 kV in the reflectron mode. Two hundred target positions were chosen for each sample using the random walk function for a total of 2000 laser shots with a power density of 18.3 kW/cm^2^.

### 2.11 Bacterial culture

*Clostridioides difficile* CCUG 37780, a nontoxigenic strain, was used in nanoparticle‐spore interaction. *C*. *difficile* VPI 10463, a toxigenic strain, was used in CDI animal model. *C*. *difficile* was cultured from stock bacteria stored at −80°C to CDC anaerobe 5% sheep blood agar (BD) in 37°C incubator under anaerobic conditions. After 48 h, colonies were transferred into brain‐heart infusion supplemented medium (BD) with 5 mg/mL yeast extract (MO BIO) and 0.1% l‐cysteine (AMRESCO®). Bacteria grow in 37°C anaerobic incubator for 2 days. The Gram‐negative bacteria *E*. *coli* O157:H7 (ATCC 35150) were cultured in Luria–Bertani broth (LB) broth and statically incubated at 37°C.

### 2.12 Purification of spores

The *C*. *difficile* spores were prepared with mixture of 70% SMC medium and 30% BHIS medium (63 g Bacto peptone, 3.5 g protease peptone, 11.1 g BHIS medium, 1.5 g yeast extract, 1.06 g Tris base, 0.7 g NH_4_SO_4_, and 15 g agar/L) in 6‐well dish, and then were incubated at 37°C under anaerobic condition (Thermo Fisher, Oxoid Ltd., Basingstoke, England) for 6–7 days. The spores were harvested from the 6‐well dish with 10–15 mL of ice‐cold sterile MQ water, vortexed for 5 min and then placed at 4°C overnight. The spore pellet was used ice‐cold sterile MQ to wash five times and centrifuged at 8000 rpm for 20 min. Then, pellet was suspended with 200 μL of ice‐cold sterile MQ. The suspension was spread on top of a 1 mL 50% (wt/vol) Nycodenz solution (Sigma‐Aldrich, St. Louis, MO) and centrifuged at 10,800 rpm for 60 min. The purified spores were washed five times with ice‐cold sterile MQ to remove extra Nycodenz solution, and stored in dark tube at 4°C.

### 2.13 Spore staining viability assay

Dropped 1 μL of purified spore solution on the glass slide and dried on air. Then, used malachite green solution (Sigma‐Aldrich, 50 g/L in water) to cover dried samples while heating the dye on heat plate for 5 min. Then, washed slide under running water and stained by Safarin O (Sigma, 5 g/L in water) for 15 s. Then, the slide was washed under the running water again. The spores showed green and the vegetative cells showed red under the light microscopy.

### 2.14 Spore viability test

Purified spores were heated at 60°C for 30 min before test. *C*. *difficile* spores were diluted in fresh sterile MQ to an optical density (OD = 600 nm) of 0.2. Then treated with 100 ppm concentration of nanoparticles for 30 min. Next, spores were germinated by adding 10 mM taurocholate acid for 12 min. Finally, samples were diluted with BHIS and spread on CDC plates. The colony forming units (CFUs) at each dilution were examined after 48 h 37°C anaerobic culture. The survival rates were calculated using the following formula: (control CFU − treatment CFU)/control CFU × 100%.

### 2.15 Spore germination assay

Purified spores were heated at 60°C for 30 min before test. *C*. *difficile* spores were diluted in fresh sterile MQ to an optical density (OD = 600 nm) of 0.2. Then treated with different concentration of nanoparticles for 30 min and centrifuged at 8000 rpm for 20 min to get the spore pellets. Finally, 90 μL BHIS and 10 μL 10 mM taurocholate acid were added for spore germination induction.

### 2.16 DPA release assay

Dipicolinic acid (DPA) release assay was measured by terbium fluorescence.^43^ Purified spores were heated at 60°C for 30 min before test and re‐suspended in germination buffer (10 mM Tris (pH 7.5), 150 mM NaCl, 100 mM glycine). Seventy‐five microliters of spore were adjusted to an optical density (OD = 600 nm) of 0.2 and then added to 96‐well white plate. Next, spore suspension is mixed with equal volume of different concentrations of nanoparticle. Then, 50 μL germination buffer containing 40 mM taurocholate and 0.4 mM TbCl_3_ was added to samples. The spores treated with TbCl_3_ only were served as a negative control. The total DPA of the spores was extracted by boiling the samples for 30 min. The signal of DPA‐terbium was monitored by FlexStation 3 Multi‐Mode Microplate Reader with excitation/emission at 270 and 545 nm, and cutoff at 520 nm.

### 2.17 Vegetative bacterial cells viability test

*Minimum bactericidal concentration* (MBC): *C*. *difficile* spores (10^4^ CFUs) were treated with 100 ppm of silver nanoparticle for 15 min. Then, samples were diluted with fresh BHIS and spread on CDC plates. The CFUs at each dilution were examined after 48 h 37°C anaerobic culture. The survival rates were calculated using the following formula: (control CFU − treatment CFU)/control CFU × 100%.

*Minimum inhibition concentration* (MIC): *E*. *coli* (10^6^ CFUs) were treated with 0–10 ppm concentration of AgAu‐based nanoboxes, and the OD600 of the bacterial suspension was measured at multiple time points using microplate reader for bacterial survival rate calculation using the following formula: (OD600_treatment_)/OD600_control_) × 100%. Ten microliters of *C*. *difficile* (10^7^ CFUs) vegetative cells were added to 96‐well plates. Then, treated with 190 μL fresh BHIS containing different concentrations of the category of nanoparticles under 37°C anaerobic conditions for 48 h. The results were observed after 2 days of culture.

### 2.18 Sustained antibacterial experiment

*Escherichia coli* (10^6^ CFUs) was daily added into 10 ppm of AgAu‐based nanoboxes and measured OD600 by microplate reader at 24 h after bacteria addition. The experiment was continuously recorded until the bacteria grew in AgAu‐containing broth. The same concentration of AgNO_3_, Ag@PVP, and Ag nanocube was also performed to monitor the sustained antibacterial time.

### 2.19 ROS determination

To determine intracellular ROS production in AgAu‐based nanobox‐treated bacteria, 5 × 10^6^ bacteria were exposed to different concentrations (0–10 ppm_[Ag]_) of AgAu‐based nanoboxes at 37°C for 30 min. After exposure, the bacteria were centrifuged and washed three times with PBS. The bacterial pellets were suspended in 100 μL of Mueller–Hinton broth (MH broth) containing 20 μM 2′,7′‐dichlorofluorescein diacetate (DCFH‐DA) for 30 min, which can passively enter into the bacteria and react with ROS to form the highly fluorescent compound dichlorofluorescein for ROS quantification. After incubation with DCFH‐DA, bacteria were washed three times and resuspended in PBS for fluorescence measurement by microplate reader with 495/520 nm excitation/emission spectra. The relative ROS level was expressed as the fold changes of fluorescence intensity compared to untreated bacteria.

### 2.20 TEM analysis of spores and vegetative cells

The interaction between the nanoparticles and *C*. *difficile* spores and vegetative cells was observed by TEM. The spores were treated with 25 ppm nanoparticles for 30 min. The samples were washed three times by sterile MQ and dropped 10 μL onto the copper grid. The samples were observed at least 4 h vacuum dried.

### 2.21 Spore integrity staining

Spore integrity was measured by LIVE/DEAD BacLight Bacterial Viability Kit. No treatment spores and treat‐nanoparticle spores were mixed with SYTO9 and PI for 15 min. The samples were observed by fluorescence microscopy.

### 2.22 CDI animal model

This study used 8‐weeks‐old C57BL/6 mice for *C*. *difficile* infected murine model. Referred to our laboratory murine protocol,^44^ mice were administrated with antibiotics mixture (kanamycin, 0.4 mg/mL; gentamicin, 0.035 mg/mL; colistin, 850 U/mL; vancomycin, 0.0045 mg/mL; metronidazole, 0.215 mg/mL; Sigma‐Aldrich) in the drinking water for 5 days before CDI. However, vancomycin and metronidazole were stopped to feed to avoid killing *C*. *difficile* on the day before infection. Then, the mice were treated with proton pump inhibitor (PPI), Esomeprazole (40 mg/kg/day; Nexium®) by five times oral gavage per 12 h interval before CDI. On the day for CDI, mice were injected with clindamycin (4 mg/kg; Sigma‐Aldrich) intraperitoneally and then treated with *C*. *difficile* spore suspension (1 × 10^8^ CFU) orogastrically.

### 2.23 Inhibition of *C*. *difficile* in a fecal bench ex vivo test

Antibiotic‐treated mice had been sacrificed; cecum content was collected and 200 μL for each tube. Then, added 50 μL *C*. *difficile* (10^7^ CFU) and 50 μL different concentrations of AgAu nanoparticles or MQ into tubes and cultured for 24 h. Next, samples were diluted with fresh BHIS and spread on CCFA plates. The colony forming units (CFUs) at each dilution were examined after 48 h 37°C anaerobic culture. The survival rates were calculated using the following formula: (control CFU − treatment CFU)/control CFU × 100%.

### 2.24 The therapeutic efficacy of the nanoparticles in CDI murine model

The CDI animal model was described above. After 48 h post‐infection, the mice were gavaged with 100 μL 500 ppm nanoparticles every 24 h for 2 days. While the control CDI mice were gavaged with sterile MQ. Mice were weighed and scored daily to monitor symptoms of CDI until sacrificed after 120 h. CDI disease progression was defined by body weight loss, colon length, cecum weight, and PCR results of *tpi* gene (specific to *C*. *difficile*).

### 2.25 The effect of the nanoparticles on animal guts microbiota

The genetic distribution ratio was estimated by real‐time PCR analysis. After normal mice had been sacrificed, cecum content was collected and treated with different concentrations of nanoparticles or MQ for 24 h. The DNA in the cecum content was extracted via the High Pure PCR Template Preparation Kit (Roche). Primers used in this study can refer to Table S1.

### 2.26 Statistical analysis

GraphPad Prism 6 was used for all statistical analyses. Values were reported as mean ± SEM. All tests in this study were done in triplicate. One‐way analysis of variance (ANOVA) and then Tukey's multiple comparison test, Student's *t*‐test were used for comparisons between groups, and differences were considered to be statistically significant with *p*‐value < 0.05.

## 3 RESULTS

### 3.1 The synthesis and characterization of AgAu‐based nanoboxes

After transferring the metallic AgAu@PSMA nanocubes^45^ into the solution containing 1.4 M H_2_O_2_ in PBS (Figure 1a), the resultant products (Figure 1b) showed cube‐like nanostructures with body‐centered diagonal lengths of nanoboxes of 61.6 nm at 0.125 mM HAuCl_4_, 65.7 ± 6.4 nm at 0.25 mM HAuCl_4_, 72.7 ± 7.2 at 0.5 mM HAuCl_4_, and 78.2 ± 7.8 nm at 1 mM HAuCl_4_, denoted as AgAu_0.125_, AgAu_0.25_, AgAu_0.5_, and AgAu_1.0_, respectively (Figure 1b). This increase in the particle size of AgAu‐based nanoboxes is due to the generation of thick wall frames of 4.9, 13.5, 17.0, and 19.1 nm, respectively, with increasing HAuCl_4_ concentrations from 0.125 to 1 mM (Figure 1b). XRD patterns (Figure 1c) demonstrated that the fractions of the AgCl crystal structure over metallic Ag‐/Au‐related composites increased with increasing HAuCl_4_ concentration. We used HADDF‐EDS line scan to prove the formation of an AgCl@Au‐rich shell, as discussed later.

As verified by the AAS measurement in Figure 1d, the Au/Ag ratio increased in the AgAu‐based nanoboxes with increasing HAuCl_4_ concentrations from 0.125 to 0.5 mM. Interestingly, the mixture of Ag@PSMA nanocubes with 0.125–0.5 mM HAuCl_4_ in the presence of H_2_O_2_/PBS resulted in the disappearance of the original SPR bands (Figure 1e) compared to their precursors (nonoxidation products; Figure S2). Notably, a new peak appeared at 256 nm, which indicated AgCl crystal formation.^46^ The far IR absorption over 1000 nm for these AgAu_0.125_ nanoboxes (Figure 1e) could be attributed to the existence of the thin‐layer AgAu nanowalls decorated with dielectric AgCl nanocrystals.^47^ AAS measurements showed that the HAuCl_4_ concentration at 1 mM did not dramatically decrease the Au count by oxidizing Ag atoms (Figure 1d), and the excess Au ions remained in the supernatant (Figure S3), suggesting that it has reached the maximum level of AgCl formation. We observed an SPR band at 711 nm for AgAu_1.0_ nanoboxes, suggesting the generation of thick Au‐based nanoshells. We further utilized a SERS experiment to demonstrate the gradual evolution of Raman signals from methylene blue for the solution mixture with the resulting nanoboxes from AgAu_0.125_ to AgAu_1.0_ (Figure 1f), proving the massive metallic nature at the surface of the AgAu_1.0_ nanoboxes.

**FIGURE 2:**
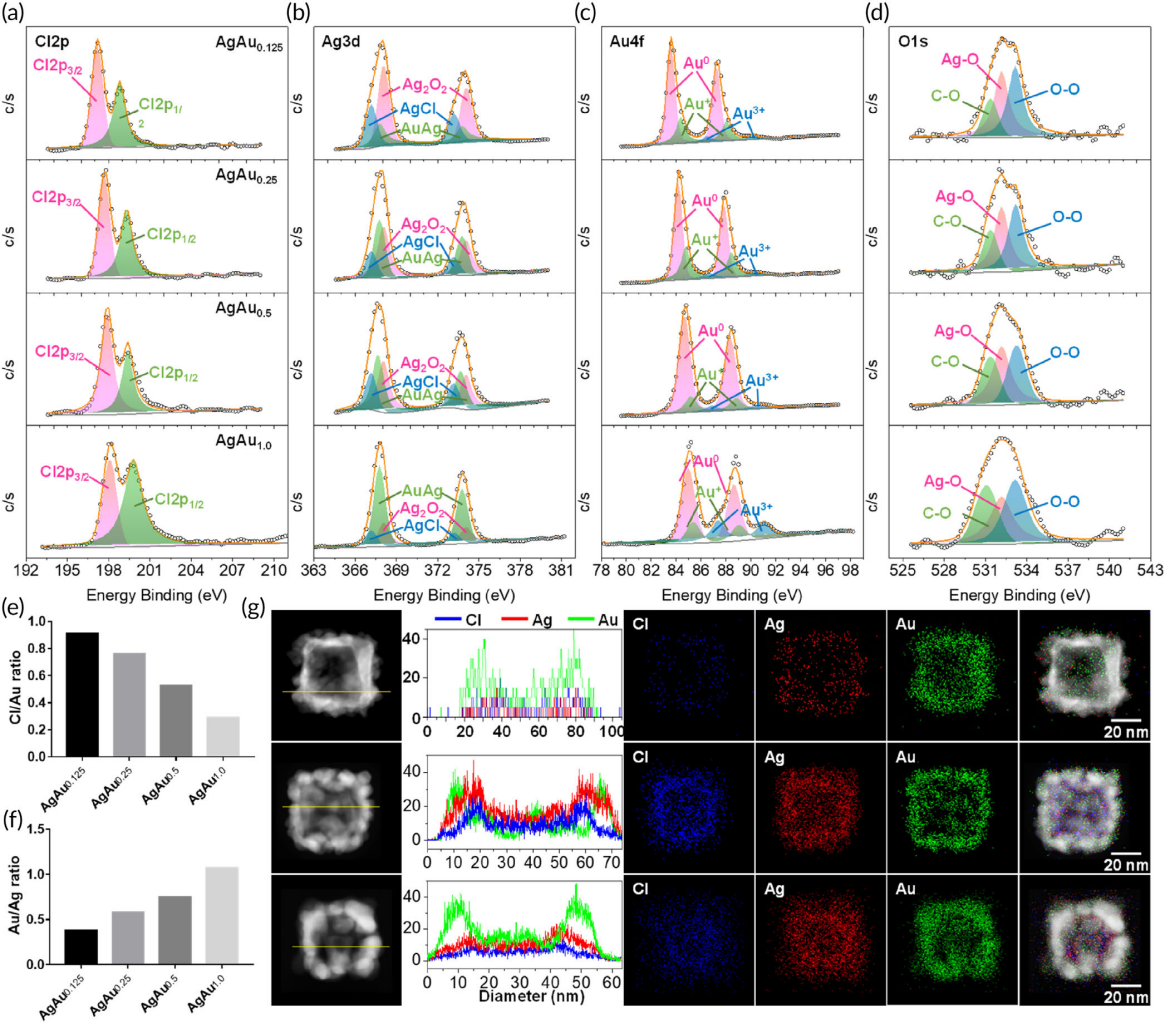
XPS measurements for the (a) Cl, (b) Ag, (c) Au, and (d) O composites on the AgAu‐based nanobox surface. The (e) Cl/Au and (f) Au/Ag ratio changes of AgAu‐based nanoboxes were calculated from the XPS results. (g) HAADF and EDS elemental line scan/mapping images of AgAu_0.125_, AgAu_0.5_, and AgAu_1.0_ nanoboxes.

The XPS spectra were analyzed to reveal the chemical states of AgAu_0.125–1.0_ nanoboxes (Figure 2a–d), and the element results in the surface analysis are presented in Figures 2e,f and S4. Figures 2e and S4a show the appearance of Cl/Au and Cl/Ag signals on the surface structure of the AgAu_0.125_ nanoboxes and were markedly decreased in the AgAu_1.0_ nanoboxes. The total Cl amount on the surface structure is 13.4%–17.4% for the AgAu_0.125–0.5_ and 4.2% for the AgAu_1.0_ nanoboxes, respectively (Figure S4b). Figure 2f shows an increase in the ratio of Au/Ag from 0.38 for the AgAu_0.125_ nanoboxes to 1.08 for the AgAu_1.0_ nanoboxes, indicating the formation of Au‐rich surface structures (Figure 1a). Consistently, the high‐angle annular dark‐field (HAADF) integrated energy‐dispersive x‐ray spectroscopy (EDS) elemental line scan and mapping (Figure 2g) of a single AgAu_1.0_ nanobox showed that the Ag and Cl atoms were primarily located in the interior area and were surrounded by the Au‐based shell. When the HAuCl_4_ concentration was decreased to 0.125 mM, the resultant AgAu_0.125_ nanowalls (Figure 2e) consisted of AgCl surface composites and AgAu nanocrystals,^48^ in agreement with the significant amount of Cl^−^ and Ag(I) ions determined in the XPS surface analysis (Figure S4b). The layer structure of the AgAu_0.5_ nanoboxes could be a possible hybrid structure of AgAu alloy and AgCl as shown in Figure 1a.

High‐resolution XPS measurements of Cl 2*p* signals at 199.5 and 196.6 eV were clearly recorded in all nanobox samples (Figure 2a). The Ag(I) ions of the AgCl crystal at 367.2 eV (Ag 3d5/2) and 373.2 eV (Ag 3d3/2) were determined in Figure 2b, which depicts a decrease in the peak areas when increasing the Au amount in the AgAu‐based nanoboxes. Intriguingly, the new Ag 3d5/2 at 367.8 eV and Ag 3d3/2 at 373.8 eV (Ag 3d3/2) are assigned to the AgOO structure.^49^ The alloy‐type Ag at 367.2 eV (Ag 3d5/2) and 373.2 eV (Ag 3d3/2)) became the predominant composition on the surface of the AgAu_1.0_ nanoboxes compared with the perfected Ag(I)Cl and AgOO in other AgAu‐based nanobox groups. It is known that the alloy type of the AgAu composition was more resistant to the oxidation and etching by H_2_O_2_ compared with pure Ag materials.^50^ However, the relative peaks of Au(III) ion species at 86.6 eV (Au 4f_7/2_) and at 90.2 eV (Au 4f_5/2_) and Au(I) ion species at 84.4 eV (Au 4f_7/2_) and at 88.0 eV (Au 4f_5/2_) could be observed and grew very well at the particle surface of the AgAu_1.0_ nanoboxes (Figure 2c). An O1*s* signal at 532.8 eV was assigned to the O—O structure in the XPS spectra (Figure 2d) in addition to Ag—O (at 531.9 eV) of AgOO^49^ and C—O (at 530.7 eV) of PSMA, as detected in Figure 2d.

Obviously, the production of the AgCl nanocrystals was assisted by reacting Ag@PSMA nanocubes in PBS/H_2_O_2_ solution with a high HAuCl_4_ concentration. It is possible that oxidizing Ag^0^ with a large amount of HAuCl_4_ led to the release of more Ag^+^ ions according to the galvanic replacement reaction,^39^, ^51^, ^52^ and then were bound to the carboxylate groups in the PSMA layer. These released Ag ions further combined with Cl^−^ ions and recrystallized in PBS solution, producing massive AgCl crystals in the XRD analysis in Figure 1c. These structures also attach to the AgAu@PSMA nanowalls (Figure 1a). Upon exposure to H_2_O_2_‐containing PBS solution, the Ag ions at the PSMA polymer also generated peroxide materials.^49^, ^53^ Note that Au(0) at 87.2 eV (Au 4f_7/2_) and at 83.6 eV (Au 4f_5/2_) was the primary derived from the AuAg alloy on the surface of the AgAu_0.5_ and AgAu_1.0_ nanoboxes. It is possible that the PSMA‐bundled and Au‐related thick shell aided in binding the metal ions^45^, ^48^ released from the dissolved nanocube templates, resulting in the difficult outward diffusion of Ag ions from the particle interior. These Ag ion species could rapidly bind to Cl ions in the PBS solution to form AgCl crystals that further attached to the inner walls of the AgAu_0.5_ nanoboxes (Figure 2g) and the AgAu_1.0_ nanoboxes. Because the Au(III) species are significantly presented on particles surface (Figure 2c) under excess HAuCl_4_ at 1.0 mM (Figure S3), the formation of Au‐based peroxide materials (Figure 2d) should originate from the homogeneous reaction between metal ions and H_2_O_2_ ^54^ at the carboxylate complex on the particle surface of the AgAu_1.0_ nanoboxes, as illustrated in Figure 1a. The peak deconvolution analyses showed a tendency of the formation of Au‐rich nanoshell structures (Figure 2c). Accordingly, the occupancy of Au‐rich structures masked the AgCl and AgOO materials at the surface structures (Figure 2b). The surface measurement in FT‐IR spectra was used to determine the vibration peaks of the O—O at 831 cm^−1^ for AgAu_1.0_ nanoboxes (Figure S5), which is located in similar positions to CaOO^55^ and the Au peroxide complex.^54^ However, due to the insufficient TEM resolution and limitation of the EDS mapping sensitivity, we were unable to identify the O elements to distinguish them on the interface of AgAu_1.0_ nanoboxes at this time, and further investigation is needed in the future.

Next, we utilized LDI‐MS to investigate the fine structures of AgAu‐based nanoboxes (Figure S6). The existence of silver and chloride species, such as Ag_2_Cl and Ag_3_Cl_2_, for all samples, would be originated from the AgCl crystals in consistence with the XRD and XPS results (Figures 1c and 2a). Consistently, the population of Ag_2_AuCl_2_ and Ag_2_Au_2_Cl species increased when the Au‐rich nanoboxes formed. The levels of Ag_2_ and Ag_3_ species decreased, accompanied by the generation of Au_3_ and AgAu_2_ molecules, as the Au concentrations increased in the AgAu nanobox structure at the same time. Besides, we found that the peroxide‐based species such as Ag_2_AuCl_2_ + H_5_O_2_, Ag_3_AuCl_2_ + H_5_O_2_, and Ag_2_Au_2_Cl + H_8_O_4_ were primarily detected in AgAu_0.125_ nanoboxes (Figure S6a). All of AgAu_0.125–1.0_ nanoboxes included Ag_2_AuCl_2_ + H_5_O_2_ peroxide composite, which co‐constructed with the alloy nanoshells at different Au concentrations, and the Ag_3_AuCl_2_+ H_5_O_2_ and Ag_2_Au_2_Cl + H_8_O_4_ disappeared when AgAu_0.25–1.0_ nanoboxes were fabricated (Figure S6b–d).

Figure S7 shows the dissolution experiment of the four AgAu‐based nanoboxes in water. The results confirmed that Ag‐ion dissolution slowed down for the AgAu_1.0_ nanoboxes, and the total released amount (2.7%) was less than that 5.1% of release rate from the AgCl crystals at 24 h (Figure S7a), indicating the formation of thick Au‐rich nanowalls that protect the AgCl nanocrystals and delay Ag^+^ release. A slow Ag^+^ ion release feature demonstrated a promising long‐term antibacterial efficacy. Notably, the concentrations of Ag^+^ in the solution at 10 min were higher for the AgAu_0.125_ nanoboxes (3.1%) than those for 2.4% by AgAu_0.5_ and the 1.8% by AgAu_1.0_ nanoboxes, which was attributed to the direct dissolution of AgCl nanocrystals from the AgAu_0.125_ nanoboxes. Compared to 2.7% of Ag ions at 24 h, the dissolution of Au was relatively low at 1.4% in AgAu_1.0_ nanoboxes (Figure S7b) due to its stable and inert structure of alloy‐based nanocomposite.

### 3.2 The rapid, efficient, and prolonged bactericidal ability of AgAu‐based nanoboxes against pathogenic *E*. *coli O157*

**FIGURE 3:**
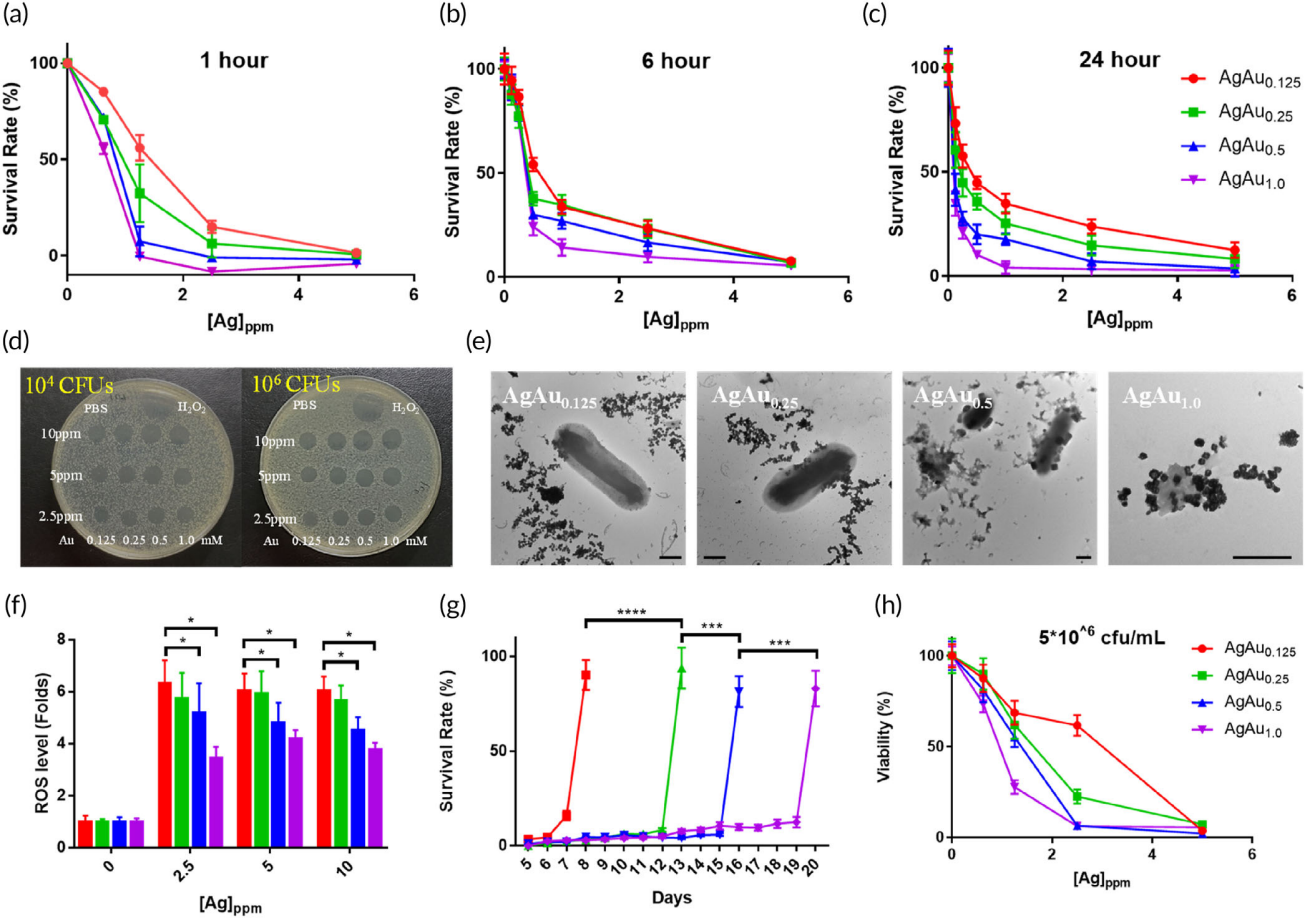
(a) 1‐h, (b) 6‐h, and (c) 24‐h bacterial survival rates upon incubated 1 × 10^6^ CFUs *E*. *coli O157*:*H7* with solutions containing the AgAu‐based nanoboxes followed by OD600 nm measurements. (d) Agar plating bactericidal assay with 10^4^ and 10^6^ CFUs bacteria treated by the AgAu‐based nanoboxes for 45 min. (e) TEM images of *E*. *coli O157*:*H7* bacteria treated by 10 ppm_[Ag]_ AgAu‐based nanoboxes for 45 min. The scale bar: 500 nm. (f) ROS production of *E*. *coli O157*:*H7* bacterial strains (1 × 10^6^ CFUs) after treatment with 2.5–10 ppm_[Ag]_ AgAu‐based nanoboxes for 30 min. (g) Sustained antibacterial activity assessed by repetitive administration of 1 × 10^6^ CFUs/mL *E*. *coli O157*:*H7* bacteria to 10 ppm_[Ag]_ AgAu‐based nanoboxes. (h) 24‐h bacterial survival rates upon incubation of 5 × 10^6^ CFUs *E*. *coli O157*:*H7* with the AgAu‐based nanoboxes then measured the OD600 nm. [*p < 0.5; **p < 0.01; ***p < 0.001; ****p < 0.0001; one‐way analysis of variance (ANOVA) followed by Tukey's multiple comparison test].

To determine the antibacterial activity of the AgAu‐based nanoboxes, we first used the clinical Shiga toxin‐producing *E*. *coli O157*:*H7* strain, which is an easier cultured enterohemorrhagic bacterial strain that growth in a facultative anaerobic condition that causes diarrhea, hemorrhagic colitis, and hemolytic‐uremic syndrome (HUS) in humans, as the target microorganism. A total of 1 × 10^6^ *E*. *coli* were incubated with 0–5 ppm AgAu‐based nanoboxes for 1–24 h (Figure 3a–c). The 1 h treatment (Figure 3a) already demonstrated dramatic antibacterial activity (10%–45% inhibition) at 0.25 ppm and a significant depletion of the bacteria at 2.5 ppm by the AgAu_1.0_ nanoboxes. As shown in Figure 3b, the killing efficiency at 1.25 ppm to *E*. *coli* upon 6 h incubation follows the order: AgAu_1.0_ > AgAu_0.5_ > AgAu_0.25_ > AgAu_0.125_. The minimum antibacterial concentrations (MICs) of the AgAu‐based nanoboxes at 6 and 24 h of incubation were 1.25 ppm (Figure 3b,c). In addition to the solution‐based antibacterial experiments, a similar tendency of highly depletive bacteria by Au‐rich nanoboxes (i.e., AgAu_0.5_ and AgAu_1.0_) could also be observed in the agar plating bactericidal assay (Figure 3d).

The TEM images showed that the AgAu_1.0_ nanoboxes, as a model group, were closely attached to the surface of *E*. *coli O157*:*H7* (Figure 3e) after 45 min of reaction time. The bacterial membrane structure became disintegrated, suggesting that the bacterial cells were seriously damaged by AgAu_1.0_ nanoboxes. Therefore, the extremely low inhibition concentration at 2.5 ppm with AgAu_1.0_ nanoboxes at 1 h (Figure 3a) could injure the bacteria, which might be due to the exposure of highly active spots at the sharp edges of the nanobox structure compared to the 1 h treatment of 10 ppm AgAu nanosphere structure in our previous work.^48^ We next performed a redox experiment with different AgAu‐based nanoboxes as catalysts for the interfacial transfer of electrons from NaBH_4_ (oxidation) to 4‐nitrophenol (reduction). As shown in Figure S8, the peroxide surface composite of AgAu_1.0_‐based nanoboxes provided a faster reaction below 3 min and complete conversion from 4‐nitrophenol to 4‐aminophenol at 30 min when compared with the static state generation of 4‐AP with the metallic AgAu@PSMA_1.0_ nanocube (without reaction in H_2_O_2_ and PBS). AgAu_1.0_ nanoboxes also exhibited efficient and superior oxidation of aminophenol to form quinone^56^, ^57^ (Figure S9). These results provided clear evidence that the delicate peroxide surface structure easily triggered redox reaction of the biomolecules in the natural state bacteria. Perhaps the outermost surface membrane of bacterial cells was oxidized when these AgAu_1.0_ nanoboxes contacted the cell surface and drew their electrons because of their strong oxidation powers of the Au(III), Au(I), and Ag(I) complex at the nanowalls,^58^, ^59^, ^60^ and specific peroxide surface structures^49^ (Figure 2a–d). The oxidized bacterial membrane of the normal microorganism would make dysfunction of the electron transport chain.^61^ To verify bacterial injury, we used DCFHDA to measure the intracellular ROS changes in *E*. *coli O157*:*H7* after coincubation with AgAu‐based nanoboxes. Figure 3f shows that all the bacteria presented high intracellular ROS levels with the four nanobox treatments in 30 min. Such high ROS production in the cell body would affect the subsequent duplication of bacteria, leading to not only reduced bacterial proliferation but also cell death.

We also demonstrated that the AgAu‐based nanoboxes have no nanozyme properties^62^ due to no superoxide, singlet oxygen, or hydroxyl radical generation when reacted with H_2_O_2_ and under 660 nm irradiation (Figure S10). These results confirm the elimination of additional injury caused by these ROS to microorganisms.

In addition, AgAu_1.0_ nanoboxes exhibited long‐term antibacterial activity compared with AgAu_0.125_ nanoboxes (Figure 3g). This activity yielded a dramatic depletion of bacteria of up to 5 × 10^6^ by using AgAu_1.0_ nanoboxes (Figure 3h). Compared with AgNO_3_ at 10 ppm with 1 × 10^6^ bacterial cells (Figure S11), our AgAu‐based nanoboxes showed a superior extended sustainable antibacterial period from 6 days (AgAu_0.125_) to 19 days (AgAu_1.0_), as shown in Figure 3g. Therefore, it was realized that AgCl crystals (Figure 1c) surrounded by the Au‐rich shell protection layer of the AgAu_1.0_ nanoboxes could prolong the amount of Ag ions by its slow and continuous in situ release (Figure S7a) from AgCl and thus possessed long‐term antibacterial activity. At concentrations of ~0.035 ppm of Au ions and 0.0675 ppm of Ag ions, based on the release rates of 1.4% Au and 2.7% Ag (Figure S7) from the 2.5 ppm AgAu_1.0_ nanoboxes, the 24 h‐MIC data showed no significant reduction of bacteria growth (Figure S12). We further removed the AgCl and peroxides on the AgAu‐based nanoboxes by washing with saturated NaCl to form dissolved metal chloride complexes. Figure S13 shows a significant decrease in antibacterial activity against *E*. *coli O157*:*H7*.

To mimic the effect of high concentrations of aminothiols in biological fluids as well as intracellular environments on the AgAu‐based nanoboxes, we pre‐incubated the nanoboxes with cellular‐abundant GSH (10 mM) for 24 h. The S 2p signals^63^, ^64^ from the Ag—S and/or Au—S bonds were detectable in Figure S14a, indicating the successful immobilization of GSH on the surface of AgAu_1.0_ nanoboxes. Figure S14b shows that the fractions of peroxide (O—O) reduced from 38.3% to 17.3% when compared to as‐prepared AgAu_1.0_ nanobox's surface structure. They lacked significant change in the fraction of Ag—O between as‐prepared AgAu_1.0_ nanoboxes (33.4%) and GSH‐passivated AgAu_1.0_ nanoboxes (35.5%). As shown in Figure S14c, we found that the GSH‐passivated AgAu_1.0_ nanoboxes weakened the antibacterial activity, which agrees with other compromised antibacterial ability of thiol‐stabilized AgNPs,^65^, ^66^ being possibly due to the hinder the bacterial membrane destruction upon the particle attachment (Figure S14d). Figure S14c shows that the AgAu_1.0_ nanoboxes perform similar tendency in killing bacteria if the GSH concentration in the incubation medium decreased to 1 mM.

### 3.3 Sporicidal ability of nanoparticles against *C*. *difficile* spores

**FIGURE 4:**
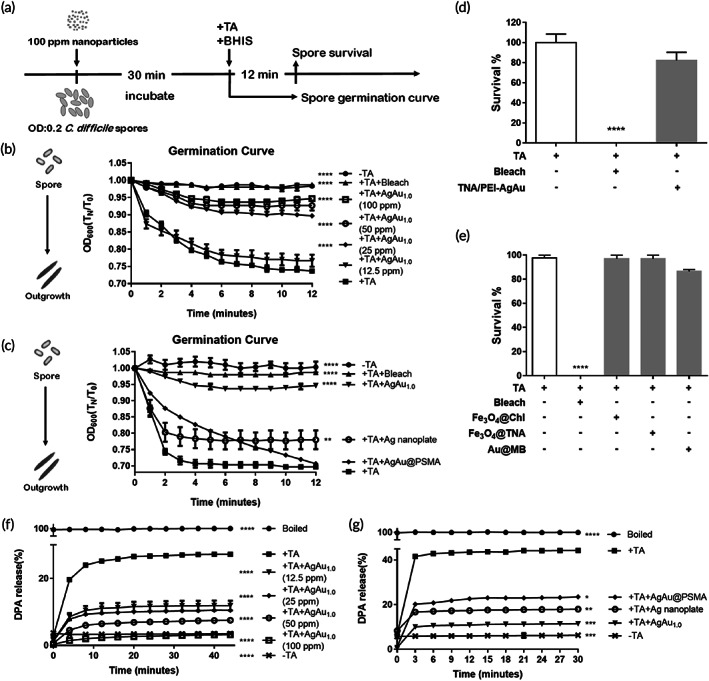
The sporicidal ability of nanoparticles against *C*. *difficile* spores. (a) Scheme of the protocol for the spore germination process and the treatment timelines of nanoparticles. The purified spores were treated with (b) different concentrations of the AgAu_1.0_ nanoboxes or (c) 100 ppm AgAu_1.0_ nanoboxes, AgAu@PSMA nanocubes and Ag nanoplate for 30 min, and then 10 mM taurocholate acid was added to trigger germination. The inhibition of spore germination by (d) positively charged TNA/PEI‐AgAu NPs and (e) PDT‐functionalized Fe_3_O_4_@Chl, Au@MB, and Fe_3_O_4_@TNA nanoparticles. The DPA release by coincubation of 1 × 10^7^ spores with (f) 12.5–100 ppm AgAu‐based nanoboxes or (g) 100 ppm AgAu_1.0_ nanoboxes, AgAu@PSMA nanocubes and Ag nanoplate. Data are the mean ± SEM [*p < 0.5; **p < 0.01; ***p < 0.001; ****p < 0.0001; one‐way analysis of variance (ANOVA) followed by Tukey's multiple comparison test].

To examine the killing effect on the harmful and elusive spores, purified *C*. *difficile* spores (~10^7^) were first prepared and then incubated with 0–100 ppm AgAu_1.0_ nanoboxes for 30 min and were then treated with TA for germination and spreading on CDC plates. Germination was measured by tracking the loss of optical density at 600 nm over 12 min at room temperature (Figure 4a). As shown in Figure 4b, spore germination was dose‐dependently inhibited by AgAu_1.0_ nanoboxes at 25–100 ppm concentrations. Compared to the 96.4% and 74.1% of spore survival by 100 ppm AgAu@PSMA and Ag nanoplate, respectively, treatment with AgAu_1.0_ nanoboxes at 100 ppm markedly suppressed the spore survival low to 8% (Figure 4c) after a 12‐min germination process. Figure 4d,e shows a lack of germination inhibition by positively charged TNA/PEI‐AgAu NPs and antibacterial TNA‐coated Fe_3_O_4_ (Fe_3_O_4_@TNA).^41^ PDT‐functionalized Fe_3_O_4_@Chl^42^ and PDT‐functionalized Au@MB nanoparticles^40^ were implemented for an additional comparison and showed insufficient or inferior suppression of *C*. *difficile* spore survival (Figure 4e). These nanoparticles were possibly not appropriate for attachment and/or caused remarkedly oxidation damage to *C*. *difficile* spores.

**FIGURE 5:**
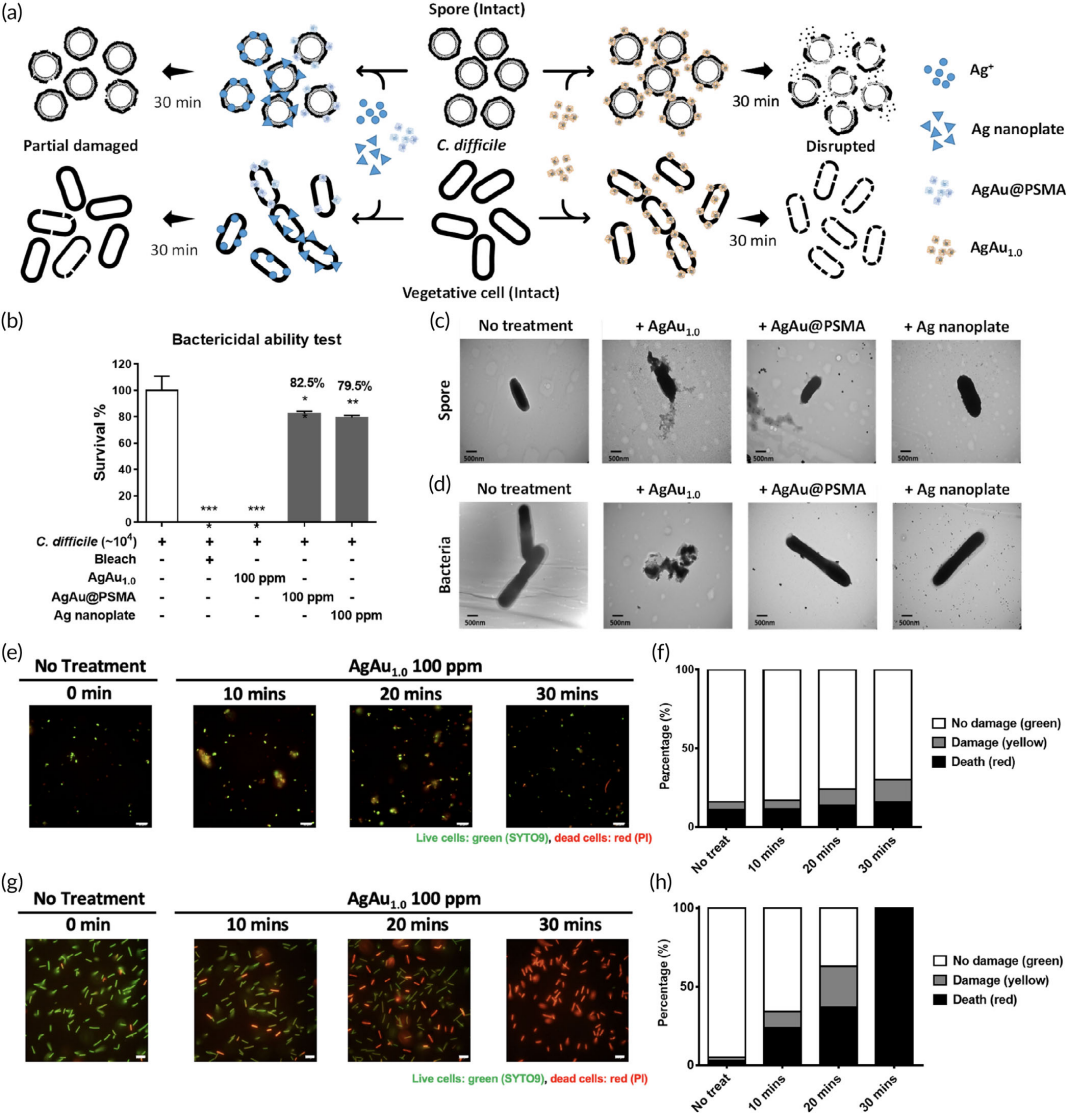
The decrease in *C*. *difficile* vegetative cell viability by AgAu‐based nanoboxes. (a) The schematic of AgAu_1.0_ nanoboxes, but not other Ag‐associated NPs or Ag ions, exhibiting bactericidal and sporicidal activity within 30 min. (b) *C*. *difficile* was treated with 100 ppm nanoparticles and then spread on CDC plates. Colony counts were determined for bacterial survival. TEM images of different silver nanoparticles interacting with (c) *C*. *difficile* spores and (d) vegetative cells by treatment with 100 ppm nanoparticles and incubation for 30 min. The AgAu_1.0_ nanoboxes damaged spore (e and f) and vegetative cell integrity (g and h) determined by a LIVE/DEAD BacLight Bacterial Viability Kit. Scale bar = 10 μm [****p < 0.0001; **p < 0.01; one‐way analysis of variance (ANOVA) followed by Tukey's multiple comparison test].

The limited sporicidal effect was further examined by measuring the release of dipicolinic acid (DPA), a dehydrated core of *C*. *difficile* spores that is biosynthesized during sporulation. As shown in Figure 4f, the fluorescence intensity at 545 nm of DPA was decreased by the AgAu_1.0_ nanobox‐treated spores in a dose‐dependent manner. This release of DPA when incubated with 12.5–100 ppm AgAu_1.0_ nanoboxes was superior, indicating the effective injurious/oxidation to the cell membrane (Figure 5a) by the particle's peroxide surface structures, when compared with the positive control group in a reaction at 100°C for 30 min (Figure 4f) and with metallic Ag nanoplates and AgAu@PSMA nanocubes at 100 ppm_[Ag]_ (Figure 4g).

### 3.4 The decrease in *C*. *difficile* vegetative cell viability by AgAu‐based nanoboxes

Figure 5b shows the bactericidal experiments on *C*. *difficile* vegetative cells (10^4^ CFUs) treated with 100 ppm nanoparticles for 15 min and then spread on CDC agar plates. Compared to Ag nanoplates (20.5%) and AgAu@PSMA nanocubes (17.5%), AgAu‐based nanoboxes exhibited higher bactericidal ability of up to 100%. We further performed MBC assay to determine the complete decrease in the viability of *C*. *difficile* vegetative cells with 25 ppm AgAu‐based nanoboxes (Table 1). The metallic AgAu_0.125_@PSMA nanocubes need a 100 ppm sample dose to approach the bactericidal effect (Figure 5b), which might be due to less destruction of the cell membrane structure in the same incubation time (Figure 5a) and its low release amount of Ag ions in the solution (Figure S15). The peroxide surface structure combined with AgCl‐based species stored in the core of the AgAu@AgCl nanoboxes was exceptionally injurious to the cell membrane and sustained Ag ion donation to interact with biomolecules of *C*. *difficile* vegetative cells. We also compared the anti‐*C*. *difficile* ability of AgAu‐based nanoboxes with metronidazole, which is the primary antibiotic used for CDI treatment. It appears that the MICs of AgAu_1.0_ and metronidazole were 6.25 and 0.5 ppm, respectively (Table S2).

**TABLE 1:** *C*. *difficile* growth inhibition by silver nanoparticles.

	Minimum bactericidal concentration (MBC) for nanoparticles against *C*. *difficile* (ppm)					P	N
	100	50	25	12.5	6.25		
AgAu_1.0_	−	−	−	+	+	+	−
AgAu@PSMA	−	+	+	+	+	+	−
Ag nanoplate	−	+	+	+	+	+	−

*Note*: The MBC of AgAu nanoparticles on *C*. *difficile* vegetative cell (10^7^ CFUs) survival.

Abbreviations: N, negative control (no *C*. *difficile*); P, positive control (*C*. *difficile* alone).

To investigate the anti‐spore and antimicrobial mechanism, TEM imaging (Figure 5c,d) was implemented to monitor the interaction between the AgAu_1.0_ nanoboxes and *C*. *difficile* spores/vegetative cells. Compared to gram‐negative bacteria, the cell wall composed of multilayer peptidoglycan of gram‐positive bacteria was thick.^67^ However, the sturdy surface structures of the spores and vegetative cells were destroyed after treating with 100 ppm AgAu_1.0_ nanoboxes for 30 min. We used a LIVE/DEAD BacLight Bacterial Viability Kit to monitor the disintegration of membrane permeability by the red fluorescent PI dye (Figure 5e–h). In the nontreated groups, spores (Figure 5e) and vegetative cells (Figure 5g) presented green fluorescence of SYTO9 and an absence of red fluorescence of PI, indicating complete membrane integrity. As the exposure time increased, PI dye infiltrated into the AgAu_1.0_ nanobox‐treated spore cores according to the evolution of red fluorescence (Figure 5f). There were still some spores without PI staining due to the strong mechanical protection structure of the spore coats. However, at the same detection intervals in vegetative cells, the fluorescent color changed from green to yellow and finally became red, and the number of red fluorescent vegetative cells obviously increased at near 100% cell death (Figure 5h). These image demonstrations directly confirmed the disruption of the membrane integrity of spores and vegetative cells by AgAu_1.0_ nanoboxes, which was illustrated in Figure 5a.

However, the ultrastructures of the spores and vegetative cells were expected to not be changed by reacting with AgAu@PSMA nanocubes and Ag nanoplates at such a short coculture period according to the relatively low bactericidal and sporicidal activity (Figures 4g and 5b–d). Obviously, these Ag‐based nanoparticles did not undergo an oxidation reaction to destroy the bacterial membrane and the spore coats (Figure 5c,d) as compared with the killing effect by the peroxide surface structure of AgAu_1.0_ nanoboxes with the strong oxidation power (Figure 5a). Considering the dissolved Ag ions examined in Figure 5d, they were not immediately accessible to inward diffusion into the cell body across the intact cell membrane of spores and vegetative cells (Figure 5a).

Before the in vivo study, the survival of *C*. *difficile* in a fecal bench ex vivo test was performed to mimic in a gut microbial community. The addition of 10^7^ CFUs *C*. *difficile* vegetative cells and 0–100 ppm AgAu_1.0_ nanoboxes in the cecum content of antibiotic‐treated mice overnight under 37°C anaerobic conditions (Figure S16a). As shown in Figure S16b, the AgAu_1.0_ nanoboxes exhibited dose‐dependent bactericidal ability: 26.9% by 12.5 ppm, 38.4% by 25 ppm, and over 93% at 50–100 ppm.

### 3.5 The therapeutic effects on CDI in vivo

**FIGURE 6:**
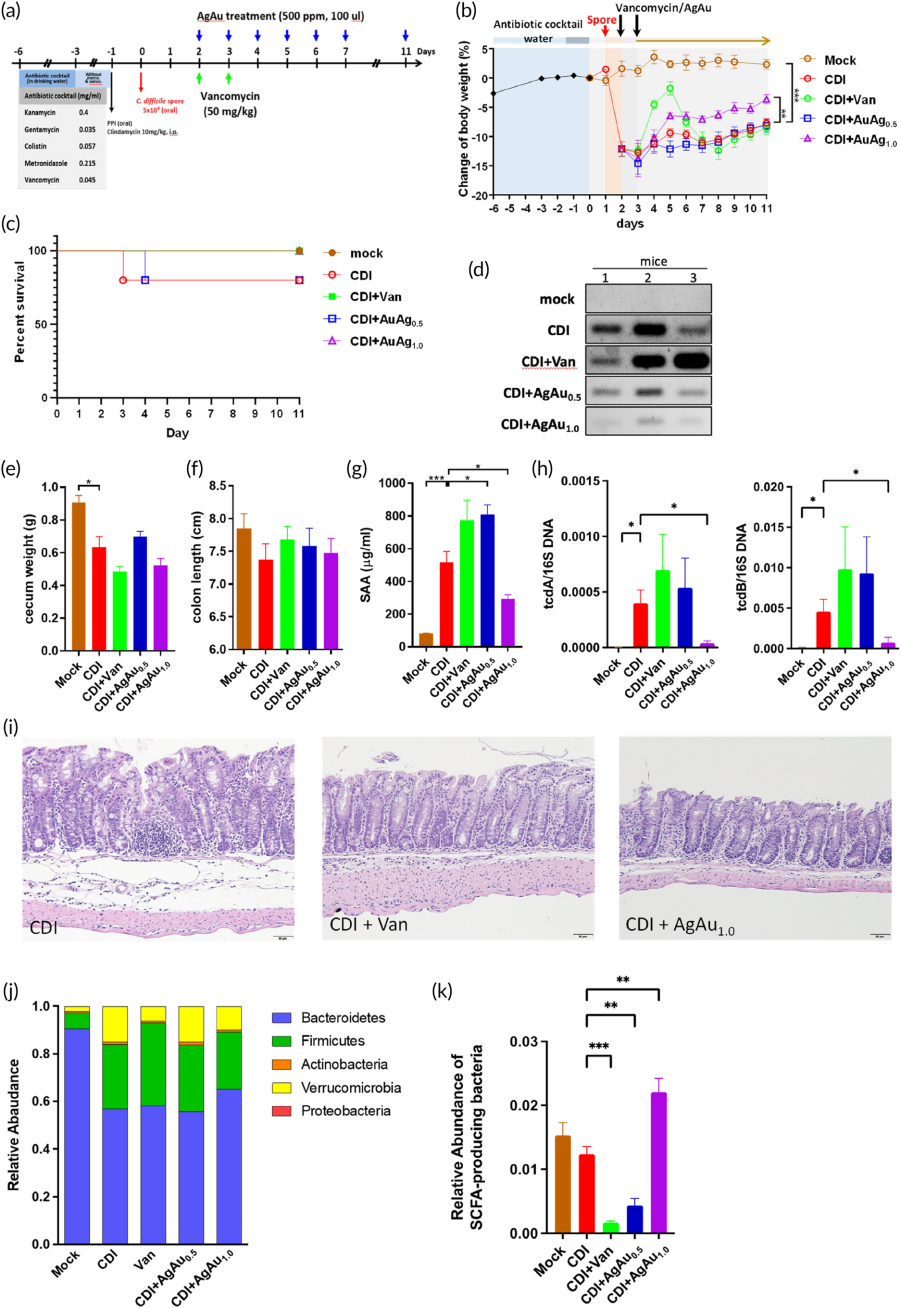
The in vivo therapeutic effects of AgAu‐based nanoboxes on CDI. (a) Scheme of protocol for CDI induction and antibiotic/AgAu‐based nanoboxes treatment timelines on murine model. The CDI disease progression is defined by (b) body weight loss and (c) survival rate; (d) *C*. *difficile* DNA determined by *tpi* PCR on mouse stool; (e) cecum weight; (f) colon length; (g) SAA concentrations; and (h) the *C*. *difficile* population determined by *C*. *difficile* Toxins A and B in the stool. (i) Microscopic examine of the colon tissues. (j) The genetic distribution ratio of colon microbiome with CDI/vancomycin/AgAu‐based nanoboxes estimated by the real‐time PCR analysis. (k) Short chain fatty acids (SCFAs) producing bacteria population quantitation [***p < 0.001; **p < 0.01; *p < 0.05; one‐way analysis of variance (ANOVA) followed by Tukey's multiple comparison test].

Based on the aforementioned successful therapeutic in vitro (Figures 4 and 5) and ex vivo results (Figure S16) against CDI, we further established a recurrent CDI murine model and evaluated disease progression. The untreated CDI group and vancomycin‐treated CDI group were added for comparisons of the enteric microflora (gut microbiota composition) in the murine infection model. At 48 h post‐oral infection of mice by toxigenic *C*. *difficile* BAA‐1805 spores, the mice were gavaged with 100 μL of 500 ppm AgAu_1.0_ nanoparticles (2 mg_[Ag]_/kg) and 50 mg/kg vancomycin every 24 h for 2 days (Figure 6a). Compared to that of untreated CDI mice, the body weight of AgAu_1.0_ nanobox‐treated CDI mice started to increase and continuously recovered to that of untreated CDI mice and the corresponding mice before CDI infection (Figure 6b). The trend of weight recovery with AgAu_1.0_ nanoboxes was slower than that of the vancomycin‐treated CDI mice but continuously increased, while vancomycin‐treated CDI mice showed recurrent CDI with a drop in body weight after Day 5. This finding suggested that the sporicidal AgAu_1.0_ nanoboxes could efficiently sustain the reversal of the CDI condition, while antibiotics can only temporally alleviate the CDI due to the insufficient sporicidal ability. The survival rate of mice was also rescued from 80% in the CDI group to 100% when treated with AuAg_1_ (Figure 6c). To examine the effect of nanoboxes on eliminating the *C*. *difficile* level in vivo, we reperformed a follow‐up experiment by detecting *C*. *difficile* genes using PCR with stool samples collected on day of sacrifice, we demonstrated three of the higher *tpi* levels per mouse group, as shown in Figure 6d and S17. The results showed a significant reduction in *C*. *difficile* residues presented in the stool samples after administering nanoboxes, especially in the AgAu_1.0_ treatment.

Regarding the clinical outcome, due to CDI recurrence, the cecum weight of the vancomycin‐treated group was lighter than that of the AuAg‐treated group on Day 11 (Figure 6e), and the colon length in the AuAg‐treated group recovered nearly to that of the mock control (Figure 6f). In addition, serum amyloid A (SAA), a major acute‐phase protein that responds to infections,^68^ was significantly decreased in the AgAu_1.0_‐treated group compared to the CDI group (Figure 6g), while vancomycin‐treated mice showed a higher infection index than that of the CDI group due to CDI recurrence. We also measured the changes in *C*. *difficile* growth, and the results showed that AgAu_1.0_ could largely reduce the *C*. *difficile* numbers compared with that of the vancomycin‐treated group (Figure 6h). The histopathological images of the colon also showed that AgAu_1.0_ nanoboxes have therapeutic efficacy by attenuating CDI through reducing inflammation and showed protection of the murine colon by preserving mucosal integrity in treated mice compared to the damage to the colon mucosa caused by vancomycin (Figure 6i). These results indicated that AgAu_1.0_ nanoboxes have better therapeutic outcomes than traditional antibiotic treatment. Although CDI recurrence was observed after Day 5 in the vancomycin‐treated group, the therapeutic outcome at Day 5 was still effective, with an increased cecum weight of approximately double that in the CDI group (Figure S18a). The colon length in vancomycin‐treated and AgAu_1.0_ nanobox‐treated CDI mice was significantly longer than that in the CDI control group (Figure S18b), and SAA was greatly attenuated in vancomycin‐treated and AgAu_1.0_ nanobox‐treated CDI mice (Figure S18c). Combined with the histopathological images of the colon that showed a recovered mucosal structure in vancomycin‐treated mice (Figure S18d), these data suggest that short‐term treatment with antibiotics can successfully reverse the CDI in mice, but there is a risk of recurrence with long‐term treatment that can be solved by alternative treatment with AgAu_1.0_ nanoboxes.

A previous study^69^ reported that DAPT‐functional gold nanoparticles have excellent antibacterial ability without considering their impact on the normal flora, which plays critical role in preventing the invasion of other pathogens in the host's gut.^70^ However, clinical treatment of CDI relies on antibiotic usage, which damages the normal flora in the human gut tract and increases the risk of CDI recurrence. Thus, an optimal antibacterial nanoparticle should be able to specifically target pathogens without disrupting the normal flora. In the long‐term assessment of the posttreatment effect on the gut microbiota, we examined the effects of AgAu_1.0_ nanoboxes on the mouse microbiome, and the genetic distribution was estimated by real‐time PCR analysis.^71^ Compared to the impact of vancomycin on the gut microbiota, the AgAu_1.0_ nanoboxes showed relatively better microbiome composition closed to normal flora while vancomycin caused worst microbiome alteration (Figure 6j). Short‐chain fatty acids (SCFAs), including acetate, propionate, and butyrate, are produced by gut bacteria through saccharolytic fermentation of complex carbohydrates. SCFAs play a crucial role in energy supply, intestinal barrier integrity, mucus production, and inflammation protection.^72^ To further understand SCFA‐producing bacterial genera, such as Bacteroides, Faecalibacterium, and Parabacteroides, real‐time PCR for analyzing SCFA‐producing enzyme genes was performed. Figure 6k shows that the administration of AgAu_1.0_ nanoboxes restored the abundance of SCFA‐producing enzyme genes. We found one of the butyrate‐producing bacteria, *Faecalibacterium prausnitzii*, was increased after AgAu nanoboxes treatment (data not shown). These findings suggest the potential beneficial effects of AgAu_1.0_ nanoboxes for treating CDI on gut health. These results revealed the potential of future application with AgAu_1.0_ nanoboxes in infected animals and humans.

### 3.6 Biosafety and biocompatibility of the AgAu‐based nanoboxes

**FIGURE 7:**
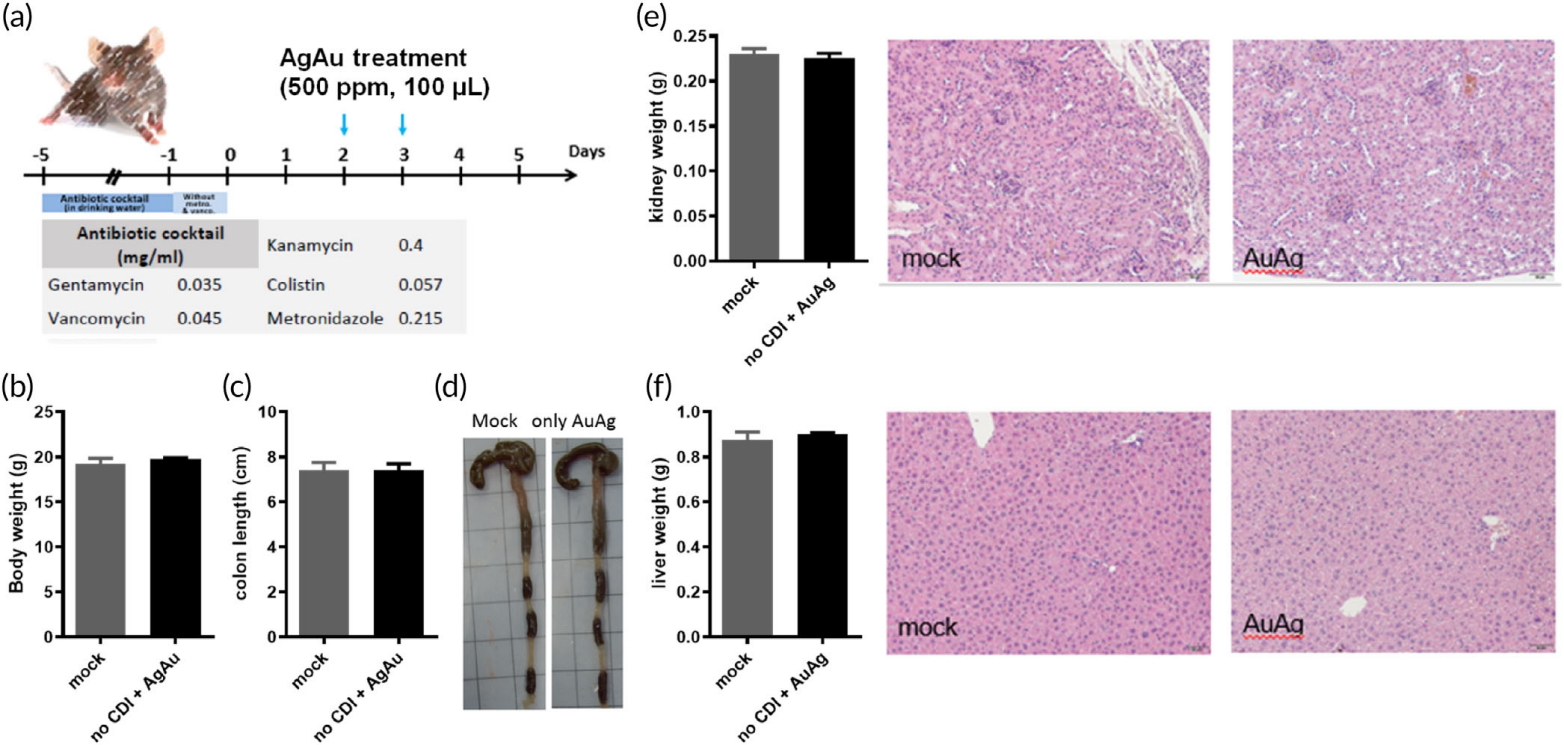
The in vivo toxicology evaluation of AgAu_1.0_ nanoboxes in non‐CDI murine model. (a) Scheme of protocol for AgAu_1.0_ nanoboxes on murine model. The (b) body weight, (c) colon length, and the (d) colon images of mice with/without AgAu_1.0_ nanoboxes. The (e) kidney and (f) liver weight and the corresponding IHC images of mice with/without AgAu_1.0_ nanoboxes. All the results were collected after sacrifice of animals on Day 5.

Notably, the biocompatibility of the AgAu_1.0_ nanoboxes was additionally studied to demonstrate the potential medical applications, especially in the cells belonging to the digestive system that would directly contact the nanomaterials. Human normal oral keratinocyte (hNOK) commonly serves as normal cell control for the toxicology test of nanomaterials and has been used extensively in biological and cancer research.^73^ The result showed that AgAu_1.0_ nanoboxes exhibited a higher cell survival rate than the AgAu_0.5_ nanoboxes with close Au/Ag ratio (Figure 1d) and those Ag‐rich AgAu_0.125_ and AgAu_0.25_ nanoboxes to hNOK cells (Figure S19), suggesting the improved biocompatibility by the exposure of Au‐rich shell structure at AgAu_1.0_ nanoboxes. In addition to in vitro test, we performed in vivo toxicology evaluation of AgAu_1.0_ nanoboxes in healthy mice (Figure 7a). The body weight (Figure 7b) and colon length (Figure 7c,d) in AgAu_1.0_ nanoboxes‐treated mice were not changed, similar to untreated animals. The kidney weight and histological examinations were the same as those of normal mice (Figure 7e), as was the liver (Figure 7f). It is worth mentioning that the mucosal integrity of the murine colon in the AgAu_1.0_ nanobox‐treated group remained normal in the in vivo study (Figures 6i and S18d).

Many previous studies of the cytotoxicity induced by Ag ions in silver nanoparticles.^74^, ^75^ Silver has been used for at least six millennia to prevent microbial infections and was the most critical antimicrobial agent available before the introduction of antibiotics.^76^, ^77^ However, its toxicity has also been a concern for modern medication. The toxicity of silver depends on its solubility and release of biologically active Ag^+^ ions; for example, acute human mortality has been observed following an abortion procedure involving the intrauterine administration of 64 mg silver/kg silver nitrate,^78^ while silver acetate has an LD_50_ of 36.7 mg/kg in mice,^79^ and silver chloride has an LD_50_ higher than 10 g/kg in mice following oral administration. For Ag nanoparticles, orally administered nanoparticulate silver was not toxic to guinea pigs at acute doses of up to 5 g/kg/day,^80^ suggesting that soluble compounds are always more cytotoxic than insoluble ones. The dosage of AgAu nanoboxes used in this study was only 2 mg/kg, which is far from the toxic threshold, and our results supported the nontoxicity to animals in vitro and in vivo. To approach proper protection, we speculated that the Au‐rich capping nanolayer at the AgAu_1.0_ nanoboxes could protect the AgCl from direct contact with the cell. These particles also exhibited a delayed release (Figure S7) and thus resisted the increased Ag ion concentration in a concise time. The proper cell variability toleration, low injury to the colon, relatively less change in gut microbiota, and superb anti‐*C*. *difficile* spores and bacteria demonstrate the practical utility of AgAu_1.0_ nanoboxes to treat *CDI*.

## 4 CONCLUSION

The AgCl/AuAg nanoshells@Ag‐based peroxide (AgAu_0.125_) and AgCl@Au‐rich nanoshells@Au‐based peroxide (AgAu_1.0_) nanoboxes were fabricated with controllable features by using low and high HAuCl_4_ concentrations in PBS/H_2_O_2_ solution. The AgAu_1.0_ nanoboxes exhibited improved bactericidal (*E*. *coli* and *C*. *difficile*) and sporicidal capabilities based on the high oxidation of the cell membrane structures in a short time. The subsequent release of Ag ions from the interior of the AgCl nanocrystals of AgAu_1.0_ nanoboxes offered an increased killing efficiency of bacteria and spores with sustainable microorganism inhibition. From the results of the in vivo study, we demonstrated that the developed AgAu_1.0_ nanoboxes could inhibit the survival of *C*. *difficile* spores and vegetative cells without causing significant colon mucosal damage and could prevent the recurrence of CDI. These AgAu_1.0_ nanoboxes possessed greatly improved biocompatibility due to the Au‐rich surface structures, which is promising for potential future translation into clinical application as a new alternative therapeutic strategy against CDI.

## AUTHOR CONTRIBUTIONS

**Li‐Xing Yang:** Conceptualization (lead); data curation (lead); formal analysis (lead); investigation (lead); methodology (equal); software (equal); validation (equal); visualization (lead); writing – original draft (lead); writing – review and editing (lead). **Yi‐Hsin Lai:** Conceptualization (equal); data curation (lead); formal analysis (lead); methodology (equal); software (lead); validation (equal); visualization (equal); writing – original draft (equal). **Chun In Cheung:** Data curation (lead); formal analysis (lead); methodology (equal); software (lead); visualization (equal); writing – original draft (lead). **Zhi Ye:** Data curation (lead); formal analysis (lead); methodology (equal); software (lead); visualization (equal); writing – original draft (lead). **Tzu‐Chi Huang:** Data curation (lead); formal analysis (lead); methodology (lead); software (equal); visualization (equal); writing – original draft (equal). **Yu‐Chin Wang:** Data curation (lead); formal analysis (lead); methodology (equal); software (equal); validation (equal); visualization (equal); writing – original draft (equal). **Yu‐Cheng Chin:** Data curation (equal); formal analysis (equal); methodology (equal); validation (equal). **Zi‐Chun Chia:** Formal analysis (supporting); validation (equal); writing – original draft (supporting). **Ya‐Jyun Chen:** Formal analysis (equal); methodology (supporting). **Meng‐Jia Li:** Formal analysis (supporting); validation (supporting); writing – original draft (supporting). **Hsiu‐Ying Tseng:** Formal analysis (supporting); validation (supporting); writing – original draft (supporting). **Yi‐Tseng Tsai:** Formal analysis (supporting); methodology (supporting). **Zhi‐Bin Zhang:** Formal analysis (supporting); methodology (supporting). **Kuan‐Hsu Chen:** Validation (supporting); visualization (supporting). **Bo‐Yang Tsai:** Methodology (supporting); validation (supporting). **Dar‐Bin Shieh:** Funding acquisition (lead); project administration (equal); resources (lead); supervision (equal); writing – review and editing (equal). **Nan‐Yao Lee:** Project administration (equal); resources (equal); supervision (equal); writing – review and editing (equal). **Pei‐Jane Tsai:** Conceptualization (lead); funding acquisition (lead); investigation (lead); methodology (equal); project administration (lead); resources (lead); supervision (lead); validation (equal); writing – original draft (equal); writing – review and editing (lead). **Chih‐Chia Huang:** Conceptualization (lead); data curation (equal); funding acquisition (lead); investigation (lead); methodology (equal); project administration (lead); resources (lead); supervision (lead); visualization (lead); writing – original draft (lead); writing – review and editing (lead).

## CONFLICT OF INTEREST STATEMENT

The authors declare no conflict of interest.

### PEER REVIEW

The peer review history for this article is available at https://www.webofscience.com/api/gateway/wos/peer-review/10.1002/btm2.10593.

## Supporting information


**Data S1:** Supporting Information.

## Data Availability

The data are available from the corresponding author upon reasonable request.
