# Supplementary material for: Novel metal peroxide nanoboxes restrain Clostridioides difficile infection beyond the bactericidal and sporicidal activity
Source: Bioeng Transl Med. 2023 Sep 5;8(6):e10593. doi: 10.1002/btm2.10593 (PMC10658501; doi:10.1002/btm2.10593)
Supplement: Supplementary file 1 — Data S1: Supporting Information. [file BTM2-8-e10593-s001.docx]

Supporting Information

**Novel metal peroxide nanoboxes restrain** **Clostridioides difficile infection beyond the** **bactericidal and sporicidal activity**

Li-Xing Yang,^a,b^† Yi-Hsin Lai,^c^† Chun In Cheung,^a^† Zhi Ye,^d^† Tzu-Chi Huang,^a^† Yu-Chin Wang,^d^† Yu-Cheng Chin,^a^ Zi-Chun Chia,^a^ Ya-Jyun Chen,^a^ Meng-Jia Li,^c^ Hsiu-Ying Tseng,^d^ Yi-Tseng Tsai,^a^ Zhi-Bin Zhang,^a^ Kuan-Hsu Chen,^a^ Bo-Yang Tsai,^c^ Dar-Bin Shieh,^b,c,e,f,g^ Nan-Yao Lee, ^h,i^ Pei-Jane Tsai^c,d,j,k*^ and Chih-Chia Huang^a,e*^

a. Department of Photonics, National Cheng Kung University, Tainan, 70101, Taiwan

b. School of Dentistry and Institute of Oral Medicine, National Cheng Kung University, Tainan 70101, Taiwan.

c. Institute of Basic Medicine, National Cheng Kung University, Tainan, 70101, Taiwan.

d. Department of Medical Laboratory Science and Biotechnology, National Cheng Kung University, Tainan, 70101, Taiwan

e. Center of Applied Nanomedicine and Core Facility Center, National Cheng Kung University, Tainan 70101, Taiwan

f. iMANI Center of the National Core Facility for Biopharmaceuticals, National Science and Technology Concil, Taipei 10622, Taiwan

g. Department of Stomatology, National Cheng Kung University Hospital, Tainan 70403, Taiwan

h. Department of Medicine, National Cheng Kung University, Tainan, Taiwan

i. Division of Infectious Diseases, Department of Internal Medicine and Center for Infection Control, National Cheng Kung University Hospital, Tainan, Taiwan

j. Research Center of Infectious Disease and Signaling, National Cheng Kung University, Tainan, 70101, Taiwan

k. Department of Pathology, National Cheng Kung University Hospital, College of Medicine, National Cheng Kung University, Tainan, Taiwan

Prof. Pei-Jane Tsai

E-mail: peijtsai@mail.ncku.edu.tw

Prof. Chih-Chia Huang

E-mail: c2huang@mail.ncku.edu.tw, huang.chihchia@gmail.com

† L-X. Yang, Y-H. Lai, C. I. Cheung, Z. Ye, T-C. Huang, and Y-C. Wang contributed equally to this work.

**Table S1. List of primers used in animal gut microbiota analysis.**

| **Microbes** | **Primer** | **Sequence (5’ to 3’)** |
| --- | --- | --- |
| *Bacteroidetes* | Bac960F | GTTTAATTCGATGATACGCGAG |
|  | Bac1100R | TTAASCCGACACCTCACGG |
| *Firmicutes* | Firm934F | GGAGYATGTGGTTTAATTCGAAGCA |
|  | Firm1060R | AGCTGACGACAACCATGCAC |
| *Actinobacteria* | Act664F | TGTAGCGGTGGAATGCGC |
|  | Act941R | AATTAAGCCACATGCTCCGCT |
| *Candidatus saccharibacteria* | Act664F | AAGAGAACTGTGCCTTCGG |
|  | Act941R | GCGTAAGGGAAATACTGACC |
| *Deferribacteres* | Defer1115F | CTATTTCCAGTTGCTAACGG |
|  | Defer1265R | GAGHTGCTTCCCTCTGATTATG |
| *Verrucomicrobia* | Ver1165F | TCAKGTCAGTATGGCCCTTAT |
|  | Ver1263R | CAGTTTTYAGGATTTCCTCCGCC |
| *Tenericutes* | Ten662F | ATGTGTAGCGGTAAAATGCGTAA |
|  | Ten862R | CMTACTTGCGTACGTACTACT |
| *Betaproteobacteria* | Beta979F | AACGCGAAAAACCTTACCTACC |
|  | Beta1130R | TGCCCTTTCGTAGCAACTAGTG |
| *Epsilonproteobacteria* | Bac960F | TAGGCTTGACATTGATAGAATC |
|  | Bac1100R | CTTACGAAGGCAGTCTCCTTA |
| *Delta- and Gamma-proteobacteria* | Bac960F | GCTAACGCATTAAGTRYCCCG |
|  | Bac1100R | GCCATGCRGCACCTGTCT |

**Table S2. List of the MIC of metronidazole, AgNO_3_ and AgAu_1.0_ nanoboxes in *C. difficile*.**

| **Compounds** | **MIC (ppm)** |
| --- | --- |
| metronidazole | 0.5 |
| AgNO_3_ | 25 |
| AgAu_1.0_ nanoboxes | 6.25 |
|  |  |

**Figure S1. (a)** The TEM image of AuAg@PSMA nanocubes. Scale bar = 100 nm.

**Figure S2**. UV-visible spectra of the AuAg-based nanoboxes without reaction in the H_2_O_2_/PBS solution at 80℃.

**Figure S3**. AAS measurements for the concentrations of unreacted Au ions in the supernatant solution after the separation of AgAu-based nanoboxes.

**Figure S4**. XPS measurements for (a) the Cl/Ag ratio and (b) the O, Cl, Au, Ag composition on AgAu-based nanoboxes surface.

**Figure S5.** The FTIR spectrum of AgAu-based nanoboxes.

**Figure S6.** The spectra of AgAu nanoboxes were obtained using laser desorption/ionization mass spectrometry (LDI-MS): (a) AgAu_0.125_, (b) AgAu_0.25_, (c) AgAu_0.5_, (d) AgAu_1.0._

**Figure S7.** (a) The Ag release results from the 100 ppm of AgCl crystals and AgAu-based nanoboxes. (b) The Au release results from the 100 ppm of AgAu_1.0_ nanoboxes.

**Figure S8.** Redox assay through the conversion from 4-nitrophenol (0.045 mM) to 4-aminophenol by using 0.25 ppm_[Ag]_ of (a,b) metallic AgAu@PSMA nanocubes and (c,d) AgAu-based nanoboxes and reacting with NaBH_4_ (25 mM). The total solution volume is 2 mL, and these sample’s absorption curve changes were recorded at 400 nm by a UV-visible spectrometer.

**Figure S9.** Redox assay through the conversion from aminophenol (2.39 M) to form quinone by using 0.25 ppm_[Ag]_ (a, b) AgAu-based nanoboxes and (c, d) metallic AgAu@PSMA nanocubes, and reacting with H_2_O_2_ (360 mM). The total solution volume is 2 mL, and these sample’s absorption curve changes were recorded at 345 nm by a UV-visible spectrometer.

To demonstrate the potential of the antibacterial capability of AgAu-based nanoboxes, ROS induction is an essential factor in killing bacteria. Once the membranes were broken, these dead cells would release H_2_O_2_ into the solution. Thus, we used the different ROS assays to determine which ROS molecules formed by reacting AgAu-based nanoboxes with H_2_O_2_, including terephthalic Acid (TA) assay for ‧OH radical, ABDA for singlet oxygen, and XTT assay for superoxide. In Figure S9a, the XTT assay has no increased absorbance at 470 nm, concerning no superoxide production. Also, the TA and RNO assays showed a negligible change in the absorption curve and absorbance from the indicators (Figure S9b-c). The three results presented that AgAu-based nanoboxes could not yield these three common ROS species by reacting with H_2_O_2_.

**Figure S10.** UV-visible and fluorescence measurements for the reaction of AgAu-based nanoboxes with 100 mM H_2_O_2_ by using (a) XTT (tetrazolium dye sodium,3′-(1-[phenylamino-carbonyl]-3,4-tetrazolium)-bis(4-methoxy-6-nitro) benzene-sulfonic acid hydrate, 0.2 mM) recorded at 470 nm of absorption in dark condition and irradiation at 660 nm (100 mW), (b) TA (terephthalic acid, 10 μM) at 440 nm of emission (excitation at 310 nm), and (c) RNO/imidazole (50 μM) at 440 nm of absorption under 660 nm irradiation for 30 min. TiO_2_ NPs (2000 ppm) with 10 min UV irradiation was used as a positive control in the XTT assay, and methylene blue (0.05 mM) was used as a positive control sample in RNO/imidazole assay.

**Figure S11.** The sustained antibacterial activity of AgNO_3_, Ag@PVP nanoparticles, and Ag nanocubes at 10 ppm with daily 10^6^ CFU of *E. coli* addition.

Based on SEM and TEM images (Figure S12a and S12b), the cell structure is rod-shaped after 30 min of incubation time with 2.5 ppm Ag ions and Au ions, indicating the minor toxicity for *E. coli O157:H7* from both metal ions to destruct the cell membrane. In addition, we studied the antimicrobial effects with MIC experiment at 0-10 ppm of Au ions. Results showed less than 20 % of the bacterial killing rate at 2.5 ppm by treating 30 min. Indeed, this short incubation time does not injure the bacteria immediately. Noted that 96.8% of bacteria die by 24h of culture time at 2.5 ppm AgAu_1.0_ nanoboxes. At concentrations of ~ 0.035 ppm of Au ions and 0.0675 ppm of Ag ions, based on the release rates of ~1.4% Au and ~2.7% Ag (Figure S7) from the 2.5 ppm AgAu_1.0_ nanoboxes, the 24 h-MIC data suggests that there is no significant reduction of bacteria growth.

**Figure S12.** The SEM and TEM (upper-right corner) images of *E. coli O157:H7* bacteria treated by 2.5 ppm_[Au/Ag]_ of (a) AgNO_3_ and (b) HAuCl_4_ for 30 min. The scale bars are 5 μm for SEM and 0.5 μm for TEM. (c) 1-hour, 6-hour, and 24-hour bacterial survival rates for the incubation of 1*10^6^ CFUs *E. coli O157:H7* with culture mediums containing HAuCl_4_ or AgNO_3_ followed by OD_600 nm_ measurements.

**Figure S13.** The bacterial viability under different concentrations of AgAu-based nanoboxes after washing using saturated NaCl solution.

**Figure S14.** Characterization and antibacterial ability of GSH-treated AgAu_1.0_ nanoboxes. XPS measurements for the analysis of the (a) S2p and (b) O1s orbitals of the 10 mM GSH-treated AgAu_1.0_ nanobox surface. (c) The 24-hour bacterial survival rates upon incubated 1*10^6^ CFUs *E. coli O157:H7* with solutions containing the AgAu_1.0_ nanoboxes, 1 mM GSH-treated AgAu_1.0_ nanoboxes, and 10 mM GSH-treated AgAu_1.0_ nanoboxes. (d) The TEM images of *E. coli O157:H7* bacteria treated by 10 ppm_[Ag]_ of 10 mM GSH-treated AgAu_1.0_ nanoboxes for 2 hour. The scale bar = 0.5 μm.

**Figure S15.** The Ag release profile of metallic AgAu@PSMA_0.125_ nanocubes.

**Figure S16**. A scheme (a) illustrated the *ex vivo* fecal bench test of the survival of *C. difficile*. (b) The corresponding experiment results of the Bactericidal ability of AgAu_1.0_ in *C. difficile.*

**Figure S17**. The original images of the *tpi* PCR results for *C. difficile* DNA determination on mouse stool. The red-box indicates the three *tpi* levels per mouse group which were demonstrated in Fig. 6d.

**Figure S18.** The therapeutic effects of AgAu_1.0_ nanoboxes on *C. difficile* infection at Day 5. CDI disease progression was defined by (a) cecum weight; (b) colon length; and (c) *the PCR-determined SAA concentrations* on mouse stool; (d) microscopic examination of the colon tissues. (**P<0.01; *P<0.05; one-way analysis of variance (ANOVA) followed by Tukey’s Multiple Comparison test).

**Figure S19.** MTT assay for the cell viability of hNOK cells under different AgAu-based nanobox treatments.
